# Geographical shifting of cholera burden in Africa and its implications for disease control

**DOI:** 10.1038/s41591-025-03847-9

**Published:** 2025-08-07

**Authors:** Javier Perez-Saez, Qulu Zheng, Joshua Kaminsky, Kaiyue Zou, Maya N. Demby, Christina Alam, Daniel Landau, Rachel DePencier, Jose Paulo M. Langa, Roma Chilengi, Placide Welo Okitayemba, Godfrey Bwire, Linda Esso, Armelle Viviane Ngomba, Nicole Fouda Mbarga, Emmanuel Wandera Okunga, Sebastian Yennan, Fred Kapaya, Stephen Ogirima Ohize, Adive Joseph Seriki, Sonia T. Hegde, Mustafa Sikder, Justin Lessler, Abhirup Datta, Andrew S. Azman, Elizabeth C. Lee

**Affiliations:** 1 Department of Epidemiology, Johns Hopkins Bloomberg School of Public Health, Baltimore, MD USA; 2 Center for Emerging Viral Diseases, Geneva University Hospitals and University of Geneva, Geneva, Switzerland; 3 Middle Distance, Portland, OR USA; 4 Instituto Nacional de Saúde, Maputo, Mozambique; 5 Zambia National Public Health Institute, Lusaka, Zambia; 6 Programme National d’Elimination de Choléra et lutte contre les autres Maladies Diarrhéiques, Kinshasa, Democratic Republic of the Congo; 7 Division of Public Health Emergency Preparedness And Response, Ministry of Health, Kampala, Uganda; 8 School of Public Health, Makerere University, Kampala, Uganda; 9 University of Yaoundé I, Yaoundé, Cameroon; 10 Ministry of Public Health, Yaoundé, Cameroon; 11 University of Douala, Douala, Cameroon; 12 World Health Organization Cameroon, Yaoundé, Cameroon; 13 Johns Hopkins Bloomberg School of Public Health, Baltimore, MD USA; 14 Disease Surveillance and Response, Ministry of Health, Nairobi, Kenya; 15 Nigeria Centre for Disease Control and Prevention, Abuja, Nigeria; 16 World Health Organization Regional Office for Africa, Emergency Preparedness and Response Program, Nairobi, Kenya; 17 The International Federation of Red Cross and Red Crescent Societies, Abuja, Nigeria; 18 The International Federation of Red Cross and Red Crescent Societies, Geneva, Switzerland; 19 Independent researcher, Rockville, MD USA; 20 Department of Epidemiology, UNC Gillings School of Global Public Health, Chapel Hill, NC USA; 21 UNC Carolina Population Center, Chapel Hill, NC USA; 22 Department of Biostatistics, Johns Hopkins Bloomberg School of Public Health, Baltimore, MD USA; 23 Division of Tropical and Humanitarian Medicine, Geneva University Hospitals, Geneva, Switzerland

**Keywords:** Bacterial infection, Policy, Research data

## Abstract

Cholera outbreaks cause substantial morbidity and mortality in Africa, yet changes in the geographic distribution of cholera burden over time remain uncharacterized. We used surveillance data and spatial statistical models to estimate the mean annual incidence of reported suspected cholera for 2011–2015 and 2016–2020 on a 20-km grid across Africa. Across 43 countries, mean annual incidence rates remained at 11 cases per 100,000 population, with 125,701 cases estimated annually (95% credible interval (CrI): 124,737–126,717) from 2016 to 2020. Cholera incidence shifted from western to eastern Africa. There were 296 million people (95% CrI: 282–312 million) in high-incidence second-level administrative (ADM2) units (≥10 cases per 100,000 per year) in 2020, 135 million of whom experienced low incidence (<1 per 100,000) in 2011–2015. ADM2 units with high incidence in central and eastern Africa from 2011 to 2020 were more likely to report cholera in 2022–2023. In hypothetical scenarios of preventive disease control planning, targeting the 100 million highest-burden populations had potential to reach up to 63% of 2016–2020 mean annual cases but only 37% when targeting by past incidence. This retrospective analysis highlights spatiotemporal instability in cholera burden and can be used as a benchmark for tracking future progress in disease control.

## Abstract (web summary)

High-resolution geospatial mapping found that the annual incidence of cholera shifted from western to central and eastern Africa between 2011 and 2020, with the latter regions more likely to report cholera in 2022–2023, reflecting instability in cholera burden patterns that can impact progress in disease control.

## Main

Cholera has long been recognized as a major global public health issue, and it remains a substantial cause of morbidity and mortality in low- and middle-income countries (LMICs). The World Health Organization (WHO) declared a global cholera emergency in January 2023, prompted by an unexpected increase in cases in both recently cholera-free and acknowledged cholera-endemic areas^1^ that reported over 800,000 cases and nearly 6,000 deaths between January 2023 and March 2024 (ref. ^2^). The emergency coincided with a prolonged shortage of oral cholera vaccine (OCV), which led the WHO to suspend the recommended two-dose course in favor of one-dose reactive campaigns, despite evidence for lower sustained protection from the one-dose regime^3^. The WHO African region has witnessed over 4,500 deaths during the emergency period and consistently experiences high cholera burden even in non-emergency periods, with roughly 100,000 of 473,000 globally reported cases in 2022 (refs. ^2,4,5^).

The 2023 emergency underscores the challenge of realizing the vision for global cholera control to which WHO member states committed at the 71st World Health Assembly in 2018 (ref. ^6^). In this 5-year interval, the Global Task Force on Cholera Control (GTFCC) and governments have made substantial progress in cross-cutting coordination, in developing and implementing guidance for the management and surveillance of cholera and its associated risk factors and in expanding the use of OCVs^7–14^. Nevertheless, additional effort is required to generate sustained progress toward global cholera control, some of which could be achieved through efficient regional targeting of control measures, such as improvements in water and sanitation infrastructure, case-area targeted interventions and mass OCV campaigns^15–19^. Current GTFCC recommendations propose to target interventions based on the past 5–15 years of incidence, and major changes in spatial burden patterns may compromise the efficiency of these plans^20^. As such, estimating the burden of disease across large geographic scales and how they change over time can be of key importance, both for tracking advancement toward broad disease control objectives^21–23^ and identifying priority areas for regional planning and coordination. Countries in Africa have led the charge in national cholera control planning, and some, including Zambia, Ethiopia and Kenya, are several years into implementation of these activities^10–13^.

Leveraging a global database of cholera surveillance data with spatial statistical models, our study presents 20-km × 20-km maps of estimated medically attended suspected cholera incidence in Africa from 2011–2015 and 2016–2020. Over this 10-year period, we examine changes in the reported overall burden, its spatial distribution and number of people living in high-incidence areas over time. In addition, we model the association between 2011–2020 cholera incidence and the spatial distribution of cholera in the post-2020 period. Finally, we assess the potential reach of targeting interventions when prioritizing by past cholera incidence.

## Results

### Mean annual incidence from 2011–2015 and 2016–2020

Our analysis dataset consisted of 30,211 distinct reports of suspected cholera cases (hereafter ‘observations’) between 2011 and 2020 from 807 distinct data sources. Observations covered 4,574 unique geographical areas (hereafter ‘locations’) and spanned seven administrative levels (863 at national levels and 29,239 at subnational levels) across 43 countries in Africa with cholera reporting for at least 1 year (Supplementary Table 1). Both time periods (2011–2015 and 2016–2020) had similar numbers of observations, but the number of unique locations and data sources was larger in the more recent period (Supplementary Table 1 and Supplementary Figs. 1 and 2). Subnational observation coverage varied considerably between countries, with the area covered by ADM2 or lower observations in at least 1 year ranging from 0.03% (Namibia) to 100% (11 countries) with a median of 85% (Supplementary Fig. 3). The analytic framework differentiated full-year and time-censored observations; 73% of observations (22,080 of 30,211) covered at least 8 months of the year and were considered full-year observations in our modeling framework (Methods and Supplementary Table 2).

**Fig. 1:**
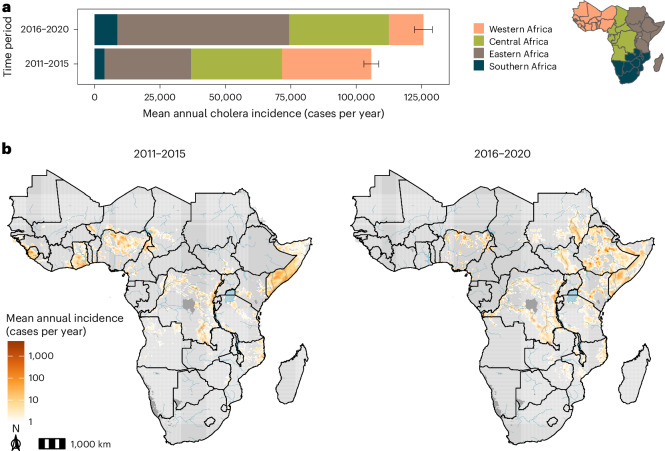
Mean annual suspected cholera incidence (cases per year) in Africa from 2011 to 2020. **a**, Mean annual suspected cholera incidence colored by region in Africa for two time periods (2011–2015 and 2016–2020). Error bars represent the 95% CrI for 4,000 posterior predictive samples of the continent-wide mean annual incidence. The colored map on the right is a legend depicting regions within Africa. **b**, Gridded 20-km × 20-km estimates of mean annual suspected cholera incidence in Africa from 2011–2015 (left) and 2016–2020 (right). Grid cells in light gray had a mean of <1 case per year, whereas those in dark gray were unmodeled due to zero population in the underlying population grid. Blue shaded areas represent major lakes and rivers in Africa.

We estimated an annual average of 125,718 (95% CrI: 122,258–129,109) suspected cholera cases across Africa in 2016–2020, which was an increase relative to 2011–2015 (105,781; 95% CrI: 102,963–108,598) (Fig. 1a). At the regional level, the 2016–2020 case burden concentrated in eastern and central Africa, whereas the 2011–2015 case burden was more evenly distributed. When overlaying the continent on a 20-km × 20-km grid, 491 million out of 1.1 billion people in 2016–2020 (355 million out of 960 million in 2011–2015) lived in areas with more than one estimated case per year (areas as measured in grid cells), but these were spatially confined to only 17% of modeled grid cells in 2016–2020 (14% in 2011–2015) (Fig. 1b and Supplementary Fig. 4).

**Fig. 2:**
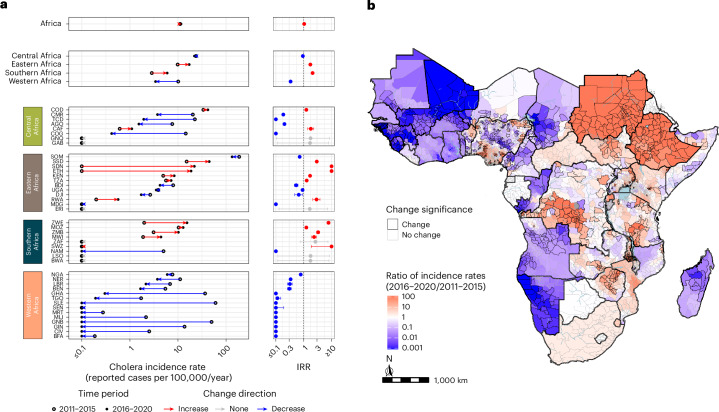
Changes in mean annual suspected cholera incidence rate (cases per population per year) in Africa from 2011 to 2020. **a**, The dot plots (left column) display changes in the posterior mean of the mean annual incidence rate from 2011–2015 (open circle) to 2016–2020 (filled circle) at the continent level (top panel), region level (second panel) and country level listed by 3-letter International Organization for Standardization (ISO3) code (four bottom panels). Arrows indicate the direction of change from 2011–2015 to 2016–2020, with red indicating increases and blue indicating decreases. Countries are ordered by decreasing 2016–2020 mean annual incidence rate within region-level panels. All changes shown in the figure were statistically significant except those in Botswana, Eritrea, Gabon, Equatorial Guinea, Lesotho and South Africa. The IRR plots (right column) display the mean (point) and 95% CrI (bars) across 4,000^2^ pairwise comparisons of 4,000 samples from the posterior distributions of the mean annual incidence rates in 2016–2020 relative to those in 2011–2015. **b**, Posterior mean ratio of the mean annual incidence rates in 2016–2020 relative to 2011–2015 by ADM2 units. Units filled with red had higher mean rates and areas filled with blue had lower mean rates in the 2016–2020 period. ADM2 units outlined in dark gray had 95% CrIs completely above or below 1, respectively. ADM2 units outlined in light gray represent statistically non-significant differences. The following countries were not eligible to have statistically significant ADM2 changes due to limited subnational data in at least one of the two periods: Côte d’Ivoire, Djibouti, Ghana, Liberia, Rwanda, Senegal, Eswatini and South Africa. Areas in light blue represent large water bodies. All areas displayed were modeled.

**Extended Data Fig. 1:**
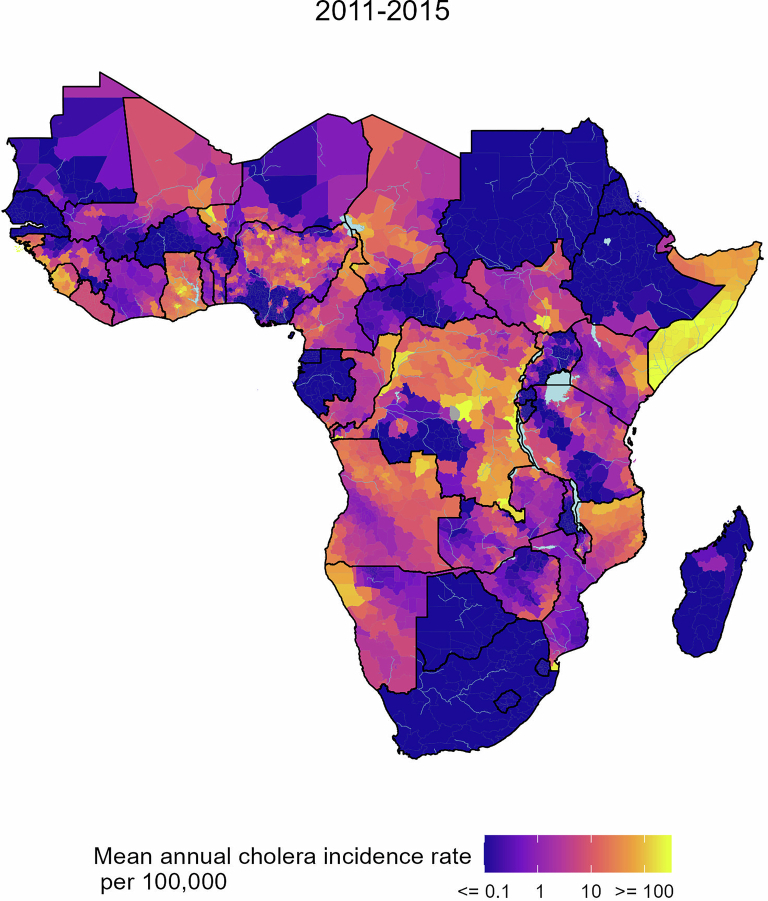
Mean annual suspected cholera incidence rate per 100,000 people at the second-level administrative units in Africa across 2011-2015. Color fill represents the mean of the posterior distribution of mean annual incidence by ADM2 unit.

**Extended Data Fig. 2:**
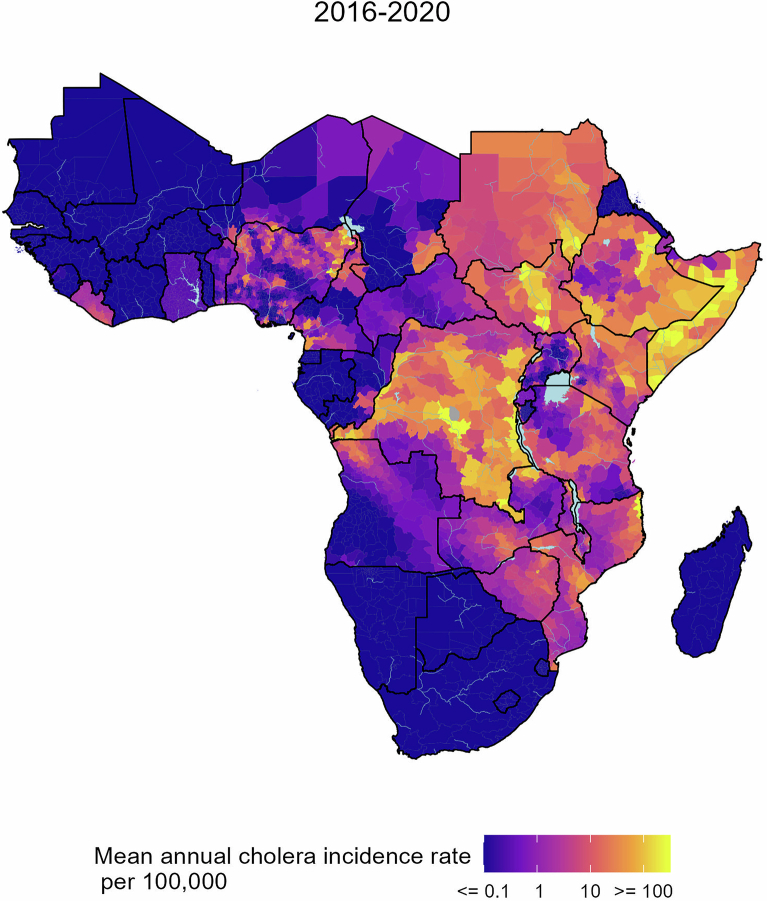
Mean annual suspected cholera incidence rate per 100,000 people at the second-level administrative units in Africa across 2016-2020. Color fill represents the mean of the posterior distribution of mean annual incidence by ADM2 unit.

The continent-wide mean annual incidence rate remained steady across both periods, hovering just above 11 cases per 100,000 population (Fig. 2a). However, there were significant regional differences, with increases in cholera burden in eastern Africa (incidence rate ratio (IRR): 1.73, 95% CrI: 1.67–1.79) and southern Africa (IRR: 2.08, 95% CrI: 1.98–2.17) and a decrease in western Africa (IRR: 0.34, 95% CrI: 0.33–0.35). Overall, 13 countries had significant increases in burden (IRR > 1) between the two periods, and 24 had significant decreases in burden (IRR < 1) (Fig. 2a). We observed subnational shifts in the spatial distribution of burden as well, including a total of 16 countries with both increases and decreases in their ADM2 incidence rates (Fig. 2b and Extended Data Figs. 1 and 2).

**Extended Data Fig. 3:**
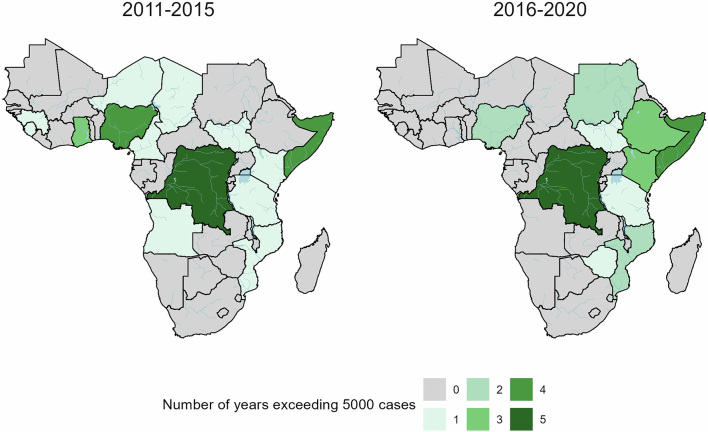
Number of calendar years exceeding 5000 suspected cholera cases in 2011-2015 and 2016-2020 by country. Countries with no years exceeding 5000 suspected cholera cases were colored in grey.

**Extended Data Fig. 4:**
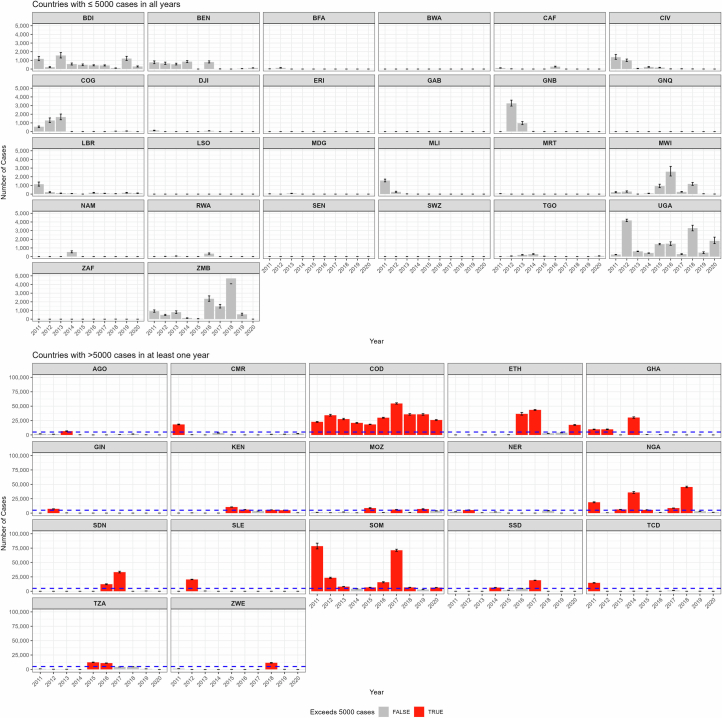
Annual country-level modeled cases by country, grouped by countries with or without more than 5000 cases in at least one year. Bar heights represent the mean and error bars represent the 95% CrI across 4000 samples from the posterior distribution. The top panel displays countries that did not have any years exceeding 5000 mean estimated cases, while the bottom panel displays countries that had at least one year exceeding 5000 mean estimated cases. Red bars and grey bars indicate years when the annual country-level modeled cases did or did not exceed 5000 cases, respectively. An absence of a bar indicates a year with less than 1 mean estimated modeled case. The following countries had less than 1 estimated modeled case in all years: Botswana, Eritrea, Equatorial Guinea, Gabon, Lesotho.

Seventeen countries in Africa had large cholera outbreaks of 5,000 or more annual reported suspected cases during the 2011–2020 decade, but the frequency of these events was highly variable by country (Extended Data Figs. 3 and 4 and Extended Data Table 1). Some countries frequently experienced substantial cholera activity; the Democratic Republic of the Congo (DRC) reported large cholera outbreaks every year, followed by Somalia in 8 of 10 years and Nigeria in 6 of 10 years. The remaining 14 countries reported large outbreaks between one and three times per 5-year period; these sporadic large outbreaks were all located in southern and eastern Africa in the 2016–2020 period, and they were dispersed across all four African regions in the 2011–2015 period.

### People living in cholera-affected areas

**Fig. 3:**
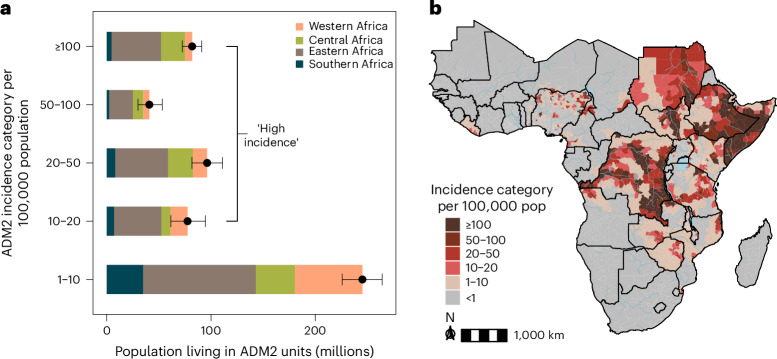
Population living in areas according to incidence category in 2016–2020. **a**, Mean (black point) and 95% CrI (black bars) for district populations living in a given incidence category (per 100,000 population) across Africa across 4,000 samples from the posterior distribution (see Methods for incidence category definitions). Regional population contributions are indicated by fill colors, and categories ≥10 per 100,000 population are labeled as ‘high incidence’ categories. **b**, Continent-wide map showing assignment of incidence categories to ADM2 units by color. ADM2 units were assigned to an incidence category if 50% of posterior draws classified the ADM2 unit to the assigned color of incidence category or above. ADM2 units in gray had an incidence category of <1 per 100,000 population. Only modeled countries are displayed in the map. pop, population.

**Extended Data Fig. 5:**
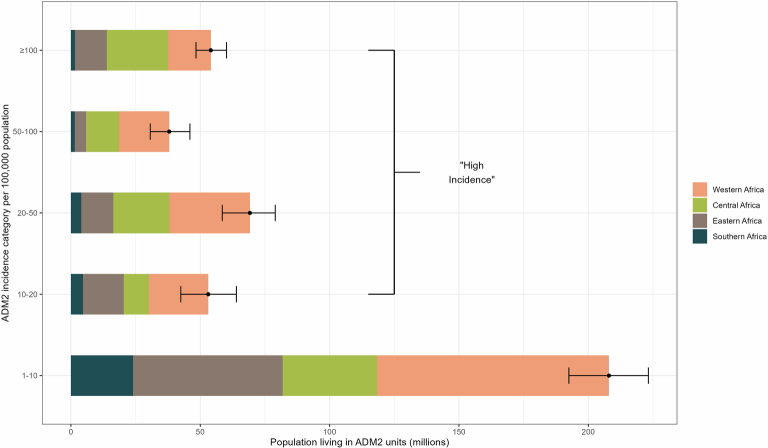
Population living in areas by incidence category and region in 2011-2015. Bar widths represent mean and error bars represent the 95% CrI of the continent-wide estimate across 4000 samples from the posterior distribution for ADM2 populations living in a given incidence category per 100,000 population. Regional population contributions are indicated by fill colors.

**Extended Data Fig. 6:**
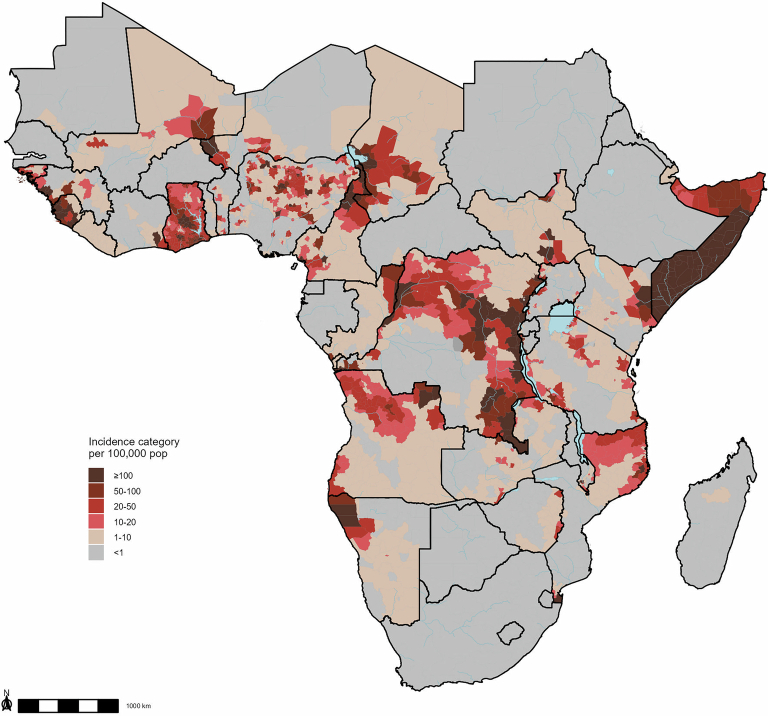
Continent-wide map showing assignment of incidence categories to second-level administrative units for 2011-2015. ADM2 units were assigned to an incidence category if 50% of posterior draws classified the ADM2 unit to the assigned color of incidence category or above. ADM2 units in gray had an incidence category of <1 per 100,000 population. Only modeled countries are displayed in the map.

In 2016–2020, we estimated that there were 296 million people (95% CrI: 282–312 million) living in high-cholera-incidence areas (≥10 per 100,000 population), among which 82 million (95% CrI: 72–91 million) experienced very high incidence (≥100 per 100,000 population) (Fig. 3a). Most people in high-incidence areas were located in either eastern Africa (166 million, 95% CrI: 155–177 million) or central Africa (66 million, 95% CrI: 60–72 million) (Fig. 3a). We found that 764 of 4,193 (18%) ADM2 units were assigned to high-incidence categories in 2016–2020 and that these were concentrated in only 20 of 43 modeled countries (Fig. 3b). Results for 2011–2015 are reported in the supplement (Extended Data Figs. 5 and 6).

**Fig. 4:**
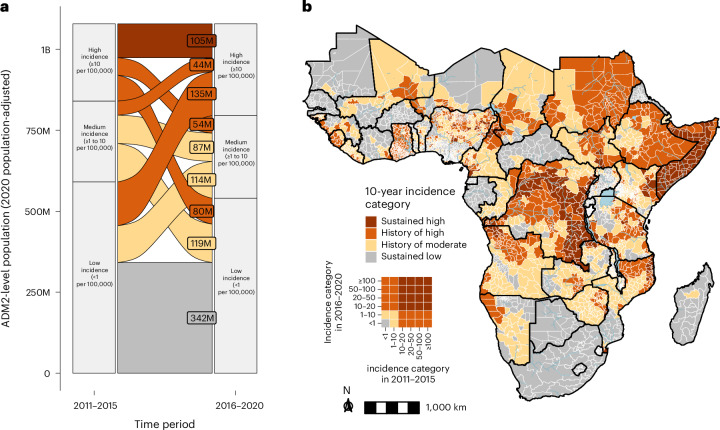
Ten-year incidence category of cholera burden in Africa across 2011–2020. **a**, Alluvial plot depicting changes in the number of people living in ADM2 units according to their 5-year incidence categories in 2011–2015 (left) and 2016–2020 (right), adjusted for 2020 population size. The flow colors indicate the 10-year (2011–2020) incidence category. The flow-specific labels indicate the number of people in ADM2 units according to their 2020 population size. **b**, Continent-wide map showing assignment of 10-year incidence categories to ADM2 units by color (see Methods for definition of 10-year incidence categories). The inset is a visual legend that translates the cross of two 5-year incidence categories into the 10-year incidence category. Gray represents ADM2 units with sustained low incidence. Only modeled countries are displayed in the map. B, billion; M, million.

**Extended Data Fig. 7:**
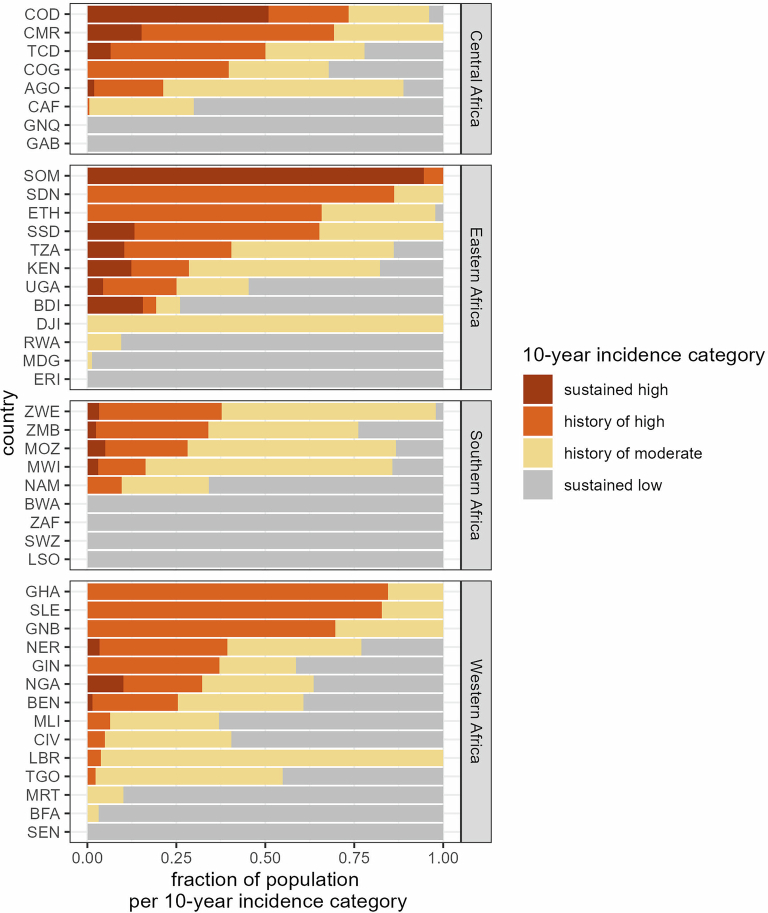
Distribution of population living in ADM2 units in each 10-year incidence category by country. Countries are grouped by region and displayed in descending order by the sum of the population fraction in the sustained and history of high-incidence categories.

Across the 2011–2020 period, we found that 105 million out of 1.1 billion people in Africa (10%) lived in 346 ADM2 units categorized as ‘sustained high’ cholera incidence (≥10 per 100,000 in both periods) (Fig. 4a), across 17 countries located mostly in eastern and central Africa (Fig. 4b). Another 313 million (29%) were in ‘history of high’ incidence (≥10 per 100,000 in exactly one period). This included people living in ADM2 units with large swings in incidence between the two periods, notably 135 million experiencing low incidence in 2011–2015 and high incidence in 2016–2020 (versus 80 million in ADM2 units shifting from high to low incidence) (Fig. 4a). Ten countries across central, eastern and western Africa had over 50% of their population living in areas with ‘sustained high’ and ‘history of high’ incidence (Fig. 4b and Extended Data Fig. 7). Overall, only 342 million people (32%) lived in ‘sustained low’ incidence ADM2 units (<1 per 100,000 in both periods) (Fig. 4a).

### Odds of cholera occurrence in 2022–2023

**Fig. 5:**
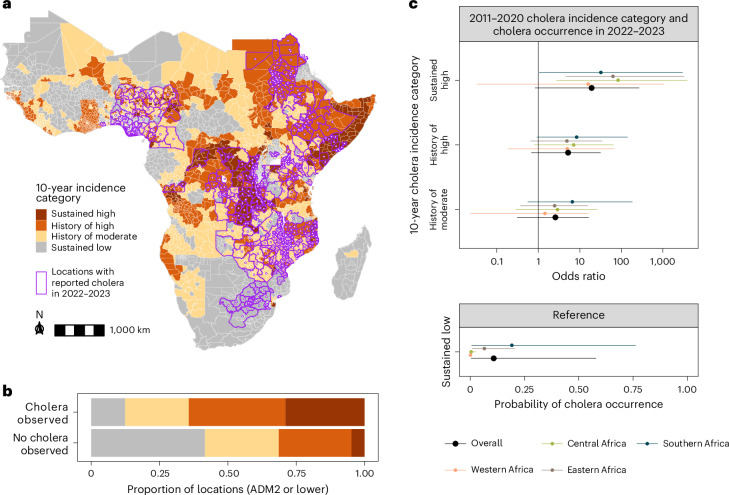
Associations between cholera occurrence in 2022–2023 and 10-year (2011–2020) cholera incidence categories in Africa. **a**, 2022–2023 cholera occurrence locations (purple borders with dots at location centroids) at the reported ADM scale overlaid on the 10-year incidence category map. **b**, Continent-wide distribution of 10-year incidence categories (colors) among ADM2 locations with reported cholera occurrence in 2022–2023. Locations with occurrence reported below ADM2 level were assigned to the corresponding ADM2 location. **c**, Pooled modeled estimates of the baseline probability of cholera occurrence in the sustained low-incidence reference category (bottom panel) and odds ratios of reporting by 10-year incidence category relative to the reference (top panel; *x* axis ticks are on the log scale, but labels are on the natural scale), at the continent level (black) and by region (colors). Points indicate mean estimates and bars indicate 95% CrIs from 4,000 samples from the posterior distribution. ADM, administrative.

**Extended Data Fig. 8:**
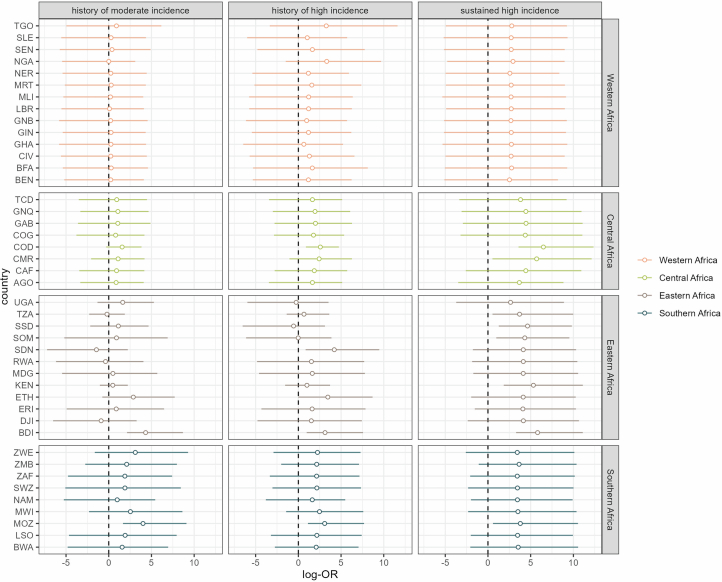
Log-odds ratios of reporting cholera occurrence in the post-2020 period by 10-year incidence category relative to the baseline probability of cholera occurrence in the sustained low incidence reference category by country. Countries are grouped by region in facets and by color. Points indicate mean log-odds ratios and error bars indicate the 95% CrIs across 4000 samples from the posterior distribution.

In 2022–2023, suspected cholera was reported in 502 geographic areas across 19 countries, among which 65.9% were at ADM2 level or lower (283 locations) (Fig. 5a). Most locations with reported cholera were in ‘sustained high’ and ‘history of high’ ADM2 units (182 of 283 ADM2 units), although 35 ‘sustained low’ ADM2 units also saw cholera cases (Fig. 5b). Using statistical models that account for possible underreporting, we found that the odds of 2022–2023 cholera occurrence tended to increase with the severity of the 10-year incidence category, although results were not statistically significant in all regions. The largest odds ratios were observed in the ‘sustained high’ category in central Africa (median odds ratio: 75.4, 95% CrI: 2.7–3,875.0) and eastern Africa (median odds ratio: 50.7, 95% CrI: 4.5–3,216.8) (Fig. 5c). Cholera occurrence was predicted to be unlikely in ‘sustained low’ ADM2 units except in southern Africa (0.19, 95% CrI: 0.01–0.76). Country-specific odds ratios were also calculated (Extended Data Fig. 8).

### Potential reach of interventions when prioritizing by incidence

**Fig. 6:**
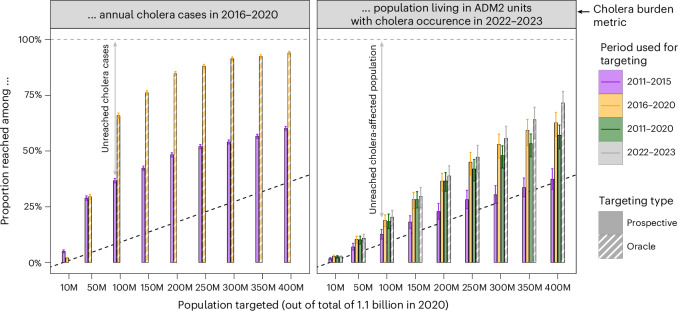
Potential reach of interventions as defined by two cholera burden metrics when prioritizing by past cholera incidence categories. Proportion of 2016–2020 cases (left panel, *y* axis) or population living in ADM2 units with cholera occurrence in 2022–2023 (right panel, *y* axis) reached when prioritizing people living in ADM2 units (*x* axis) by past incidence categories (‘prospective’ targeting, full bars) or burden in the concurrent period (‘oracle’ targeting, hashed bars). There is only one past incidence category (2011–2015) for the 2016–2020 period. There were three past incidence categories (2011–2015, 2016–2020 and 2011–2020) for the 2022–2023 period. The horizontal dashed line marks 100% of cases or population reached. The diagonal dotted line indicates unit yield in population targeted (for example, targeting 10% of the population in Africa reaches 10% of cases in 2016–2020 or cholera-affected population in 2022–2023). Bar heights represent the mean and error bars represent the 95% CrI of the mean estimate across 4,000 samples from the posterior distribution. M, million.

**Extended Data Fig. 9:**
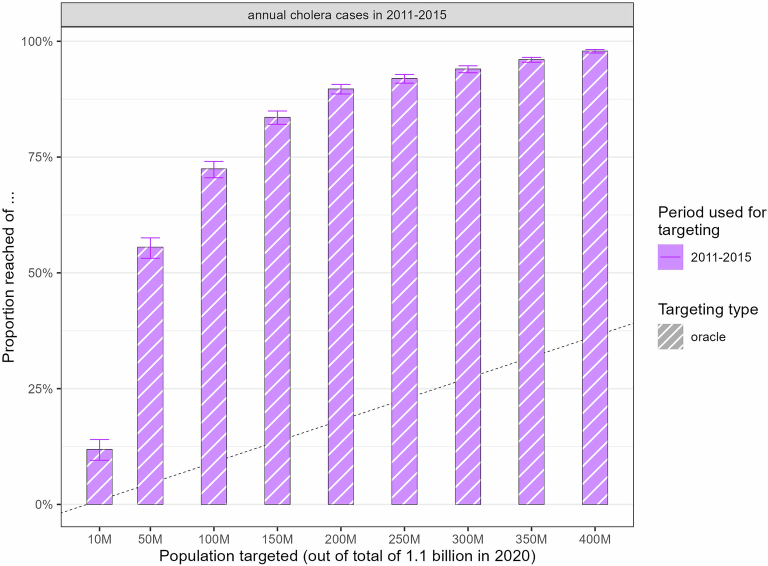
Proportion of 2011-2015 cases reached when prioritizing people living in ADM2 units by 2011-2015 incidence categories. The y-axis indicates the proportion of 2011-2015 cases reached at different number of people targeted with hypothetical interventions across the continent (x-axis) according to “oracle” targeting. Bar heights represent the mean and error bars represent the 95% CrI of the mean estimate across 4000 samples from the posterior distribution.

As guidance for long-term preventive cholera control planning recommends geographic targeting of interventions such as vaccination^20^, we investigated the potential population reach when prioritizing intervention targets by incidence. Due to spatial clustering of areas with high burden, targeting interventions to ADM2 units based on incidence categories would theoretically enable potential reach to a greater proportion of cases than proportion of population targeted. For instance, assuming that incidence categories are known (‘oracle’), targeting the top 50 million people in the highest-burden areas (roughly 5% of total population) would reach 29% (95% CrI: 28–31) of 2016–2020 cases and 66% (95% CrI: 65–67) when targeting the top 100 million (10% of total population) (Fig. 6), with similar or better yields in 2011–2015 (Extended Data Fig. 9). Using 2011–2015 patterns for planning 2016–2020 interventions (‘prospective’) achieved yields similar to ‘oracle’ targeting only for the top 50 million people in the highest-burden areas and had significantly reduced potential intervention reach beyond. For example, targeting the top 100 million of the highest-burden population in 2011–2015 would reach only 37% (95% CrI: 36–38) of 2016–2020 cholera cases. Furthermore, we found that using more temporally distal incidence categories for prioritization decreased potential reach of interventions applied to locations with cholera in 2022–2023. Targeting the top 100 million people based on 2016–2020 incidence categories reached 19% (95% CrI: 16–21) of the 2022–2023 cholera-affected population versus 13% (95% CrI: 11–15) using 2011–2015 incidence categories, with an increasing difference in yield as more people were targeted (Fig. 6). Using the 2011–2020 incidence categories for targeting performed similarly to targeting based on 2016–2020 incidence categories.

## Discussion

By developing high-resolution maps of cholera burden in Africa, we highlight challenges in making progress toward cholera control, given that cholera burden in the continent, although persistent, demonstrates shifting patterns across space and over time. We found that reported suspected cholera cases increased in 2016–2020 relative to 2011–2015, although the mean annual incidence rate remained stable across the two periods. Western Africa experienced a sharp decline in 2016–2020 relative to 2011–2015, which was offset by increases in cholera in eastern Africa. We also saw geographic shifts at the subnational level in 16 cholera-affected countries. Nevertheless, consistent with a previous analysis^4^, cholera burden remained spatially concentrated, with only 18% of ADM2 units having high cholera incidence in 2016–2020. The 82 million people living in areas with very high incidence and the 296 million living in high-incidence areas in 2016–2020 could be viewed as a shortlist of potential spatial targets for high-impact disease control investments while also highlighting a large potential OCV demand that has consistently exceeded past and projected global production capacity (37–50 million doses in 2024)^24^.

The regional shifts in mean annual incidence are but a simplified view of distinct local and regional outbreaks, changes in reporting and underlying risk factors. In western Africa, widespread cholera transmission occurred from 2011 to 2015, with notable outbreaks in 2011, 2012 and 2014 in coastal countries such as Guinea-Bissau, Guinea, Ghana, Sierra Leone, Benin and Nigeria^25^. From 2016 to 2020, however, reported cholera cases in the region declined sharply, with most cases confined to Nigeria. In contrast, eastern Africa experienced a significant increase in Ethiopia and Sudan in 2016 and 2017 after minimal activity in the 2011–2015 period, although some part of this increase may be explained by expanded data availability in more recent years; for example, cholera surveillance in Ethiopia became centrally reported and, thus, more readily available in 2015 (ref. ^26^). Beyond reporting, a complex web of factors may have also contributed to underlying epidemiologic changes across Africa during this period. Although improving, population access to clean water and sanitation remains limited in many areas, and conflict, natural disasters and other climatic and contextual factors can exacerbate conditions that enable cholera transmission^27,28^. For example, population displacement was thought to have driven spatial cholera patterns during the 2014–2017 outbreaks in South Sudan, and climatic events that contribute to water scarcity and water contamination have been posited as drivers of 2016–2017 outbreaks in Ethiopia^29,30^. On the other hand, the devastating 2014–2016 Ebola outbreak in Guinea, Sierra Leone and Liberia^31^ may have triggered population behavior changes that had the unintentional effect of reducing cholera risk in these countries^32^. Nevertheless, beyond these examples, the attribution of shifting cholera burden across Africa to specific factors remains an open question requiring further investigation.

Geographic shifts represent only one facet of the instability in cholera burden across Africa; whereas some areas sustained high incidence from 2011 to 2020, even more areas experienced it only sporadically. There were 105 million people living in ADM2 units with high incidence throughout 2011–2020 who also experienced higher odds of cholera in 2022–2023. However, these 105 million constituted only a quarter of people who experienced any high incidence during the decade, and this figure is dwarfed by the number experiencing extreme shifts during this time (for example, areas that were low incidence in 2011–2015 and high incidence in 2016–2020 were home to 135 million people). This instability in spatial patterns continued into 2022–2023, where we estimated an 11% probability of cholera occurrence among ADM2 units sustaining low cholera incidence from 2011 to 2020 (driven mostly by outbreaks in southern Africa). Further instability is evidenced by the 2024 reemergence of cholera in Ghana after 8 years without outbreaks^33^. These observations suggest that populations without recent cholera activity remain vulnerable to reintroduction without widespread and stable improvements in water and sanitation access. Genomic analyses have repeatedly suggested that long-range introduction of the cholera-causing bacteria *Vibrio cholerae* plays a critical role in cholera transmission in Africa, as introduced lineages tend to circulate clonally, sometimes for decades, in distinctly separate geographic subregions of western and eastern Africa^34–36^. Consequently, we hypothesize that geographic instability is a key feature of cholera epidemiology in Africa. In this context, the sporadic and spatially clustered nature of cholera burden may be explained by a complex interplay of infrequent introductions of *V. cholerae* into a region, population movement within that region, underlying socioecological risk factors of transmission and the population immune landscape.

The spatiotemporal clustering of cholera incidence may be leveraged to identify where interventions can be most efficiently deployed, particularly in the face of declining resources for global health and limited OCV supply^7,15^. Although targeting the top 10% of 2016–2020 populations with highest burden would reach over 60% of cholera cases in that period, shifting spatial patterns mean that this yield decreases significantly when using past patterns to target future interventions (for example, targeting the top 10% of 2011–2015 highest-burden populations would reach only 37% of 2016–2020 cases). This suggests that when countries identify priority areas for multisectoral interventions (PAMIs) for national cholera planning following GTFCC recommendations^20^, the robustness of PAMI selections should be evaluated across multiple time ranges and consider changing epidemiologic context and risk factors. Nevertheless, our results suggest that targeting populations with recent very high incidence (≥100 per 100,000 population in the last 5 years) or high incidence over sustained periods (≥10 per 100,000 population over 10 years) has the best potential to maximize the efficiency of cholera intervention reach.

This study has several limitations. Interpretation of the magnitude and spatial distribution of these maps is limited by the underlying data, which comprise medically attended suspected cholera across multiple case definitions and can vary adaptively by transmission setting and location^37^. As previously mentioned, apparent increases in burden or changes in spatial patterns in our estimates could be highly sensitive to changes in reporting and data availability (Supplementary Figs. 1 and 2). Although we know that there is high variability in the proportion of suspected cases truly caused by *V. cholerae*, estimates of reported suspected cholera are what is primarily being used to track progress and target interventions on large regional scales. We expect to see improvements in the estimation of true cholera incidence in the coming years with expanding rapid diagnostic test usage and clearer testing guidance^9,37^, and continued efforts to sustain high-quality surveillance are needed to better characterize long-term changes in cholera epidemiology. Nevertheless, our maps and modeling methodology improve upon previous estimates of cholera burden in Africa due to substantial enhancements to spatial data coverage, data processing and modeling fidelity^4^. We further note that we did not account for additional external drivers of reported cholera in Africa, such as changes in case reporting due to policy or due to the COVID-19 pandemic in 2020. Another simplifying assumption of our modeling framework is that the spatial patterns of cholera incidence remain stable within each time period. However, we account indirectly for reporting variability through overdispersion in the observation process at subnational scales. Finally, as we modeled each country separately, we did not enforce cross-border consistency in mean annual incidence estimates. In some regions, smooth estimates were nevertheless recovered from subnational data, but other borders presented discontinuities that consist of opportunities for further research (Methods).

The stability in continent-wide cholera incidence rates and the resurgence of cholera in previously low-burden areas may be disheartening to those who know how much effort has been invested in cholera control during this period. What remains clear is that multisectoral control efforts and research must expand and accelerate to offset the forces limiting measurable progress. Our continent-wide analysis serves as a benchmark for cholera control by providing important regional context, serving as a reference for large outbreak events (Extended Data Figs. 3 and 4 and Extended Data Table 1) and supplementing country analyses to identify priority intervention areas, which incorporate local knowledge of risk factors and surveillance gaps at more resolved spatial and temporal scales^29,30,38–48^. Regular high-level mapping analyses such as this are, therefore, critical to keeping the global cholera response relevant and to tracking progress toward disease control goals.

## Methods

### Cholera incidence data

#### Data sources

We curated a global cholera incidence database of national and subnational surveillance data and other reports from ministries of health (MOHs), GTFCC partners and other public data sources for countries in Africa from 2011 through 2020, which included a comprehensive online search for national and subnational cholera outbreak reports for every modeled country and year. Shapefiles were obtained from MOHs, WHO country or regional offices, unified or curated sources such as GADM, geoBoundaries and GRID3 and other online sources (https://data.humdata.org/ and ref. ^49^) and were linked to observation locations. Suspected cholera case definitions varied by data source and were oftentimes not stated but were commonly variations of the recommended WHO suspected case definition, such as ‘any patient presenting with or dying from acute watery diarrhea’ and ‘a patient aged 2 years or more develops acute watery diarrhea with or without vomiting’ (see Supplementary Table 3 for a complete list).

All countries in Africa that had at least one national-level report of suspected cholera (including zero) in both periods of analysis were modeled (Supplementary Table 1). Following this criterion, 11 of 54 countries in Africa were excluded (Algeria, Cape Verde, Comoros, Egypt, Gambia, Libya, Mauritius, Morocco, Sao Tome and Principe, Seychelles and Tunisia).

#### Data collection protocol and data template

All cholera surveillance and alert documents were systematically scraped for all reported counts of suspected cholera (‘observations’) that were explicitly linked to date ranges and geographical areas (‘locations’) and were thought to represent all cases reported in a specific space-time unit (for example, not representing just a subset of cases, such as age-statified or sex-stratified counts).

Documents were extracted only when contextual information suggested that the document creator thought the data represented a real cholera outbreak. For regularly updating data sources (for example, situation reports), older data were updated to the most recent back-corrected case counts to limit issues stemming from incorrect initial ascertainment and reporting delays and irregularities. Prior to running our final set of models, we also conducted a data audit on highly discrepant outlier observations (for example, sum of subnational case counts greatly exceeded national case counts and high variation in case counts during roughly the same period) to correct individual observations and prune likely reporting errors on a case-by-case basis.

Location names were systematically verified, and associated geographic shapefiles were identified with a standard location audit protocol, which consisted of searches on reputed websites and resources and comparison to locations that already existed in the Cholera Taxonomy database^4,50^. Metadata, source documents, shapefiles and observations were then added to the global cholera surveillance database. Each observation contained the following information: location shapefile, date range, number of suspected cases and time fraction (tfrac) within a calendar year, which is calculated from the date range.

### Cholera data processing

Cholera data were extracted from the database and passed through a processing pipeline to format and harmonize raw data inputs (which covered a wide range of temporal and spatial scales) for our statistical mapping modeling framework (Supplementary Fig. 5). The main data processing steps consist of temporal aggregation to the yearly timescale, identifying temporally censored observations (those spanning fewer than 8 months of a year), filtering observations that do not contribute to the likelihood, imputation of limited national-level observations and assigning observation-linked geographic areas (‘locations’) to the spatial modeling grid. After modeling, the resulting gridded estimates undergo postprocessing to produce estimates for unified, non-overlapping administrative units.

#### Temporal aggregation

Our statistical mapping model aimed to infer mean annual incidence rates, so we sought to aggregate observations to the annual time resolution. As observations may exist for arbitrary locations and date ranges, non-overlapping observations that were consecutive in time were aggregated if they were in the same location, calendar year and source document. If a location had multiple observations of suspected cholera for the same time bounds within the same data source, we included the observation with the largest case count in the aggregated observation; the implicit assumption here is that cases are more likely to be underreported than overreported, so we give preference to higher case count reports. This resulted in a set of aggregated observations per location, year and data source and the corresponding fraction of calendar year that they covered, which were used as model inputs.

#### Identifying time-censored observations

Our modeling framework did not assume that cholera incidence was homogenous throughout the year (see Methods, ‘Statistical framework for modeling mean annual incidence’), and we, therefore, differentiated full-year observations from partial-year observations in the model likelihood. Partial-year observations were considered to be right-censored if they spanned fewer than 8 months (0.65 years). We dropped all right-censored observations with zero suspected cases, as these have a likelihood of 1 and, therefore, do not contribute to the model likelihood.

#### Observation filtering

Observations were dropped from inclusion in the model if they have a likelihood of 1 (and, therefore, would not contribute to the likelihood) or were otherwise not informative to the model. Although some of these decisions are discussed elsewhere in Methods, broadly speaking, observations that were removed include those that (1) were not associated with a geographic shapefile, (2) were ADM0 (country-level) observations that spanned multiple years, (3) exactly duplicated observations prior to temporal aggregation, (4) had the exact same location and time bounds but reported fewer cases than another temporally aggregated observation from the same data source, (5) were time-censored observations that reported zero cases, (6) were time-censored observations at the ADM0 level that had less than half the reported cases as full-year ADM0 observations in that timeslice or (7) had zero population according to the WorldPop raster.

#### National-level data imputation

At least one country-level annual observation was sought for every country–year combination modeled to improve model stability and performance. This imposed a critical constraint to bound modeled incidence rate estimates, particularly when fitting a model to data with only censored observations or only subnational observations and incomplete spatial coverage across the country.

When a country-level observation was not found in a given year, imputation of a country-level annual observation was performed. If no observations at any spatial scale were available for that year, a zero-case observation was imputed, thus assuming that absence of data in a year corresponded to a report of zero cases for that country. If subnational observations were available and they covered a non-overlapping spatial area that represents at least 10% of the country population, a mean tfrac-adjusted incidence rate was computed across all subnational observations and multiplied by the country population to impute a country annual observation. If subnational observations were available and they covered a spatial area representing less than 10% of the country population, an observation was imputed as the maximum of the sum of cases across all unique data source and administrative unit level combinations. If only censored national observations and no subnational observations were available, the maximum value censored observation was imputed.

In the end, a limited number of observations were imputed (109 imputed relative to 30,102 non-imputed observations) in order to ensure that all modeled countries had at least one country-level observation per year. Of these, zero-case observations were added when no annual country-level report was found (96 imputed observations in 21 countries). When subnational or censored national annual reports were available, non-zero-case observations were aggregated to impute an annual country-level report (13 imputed observations in 10 countries).

#### Geographic linkage of observations to modeling grid

Our statistical modeling framework was applied to a space-time modeling grid, where the space dimension was composed of 20-km × 20-km grid cells that overlapped with a given country’s geographic shapefile and had a population greater than zero according to the associated WorldPop gridded population estimates in that year; the time dimension was represented in annual time slices. In our cholera surveillance database, some observations corresponded to an area roughly the size of a 20-km × 20-km grid cell, but very few were smaller than that. We, therefore, selected the 20-km × 20-km grid scale as a compromise between the limits of inference in the available data, computational tractability and spatial granularity of the burden estimates, following the example of previously published cholera burden maps^4^.

Observations of suspected cholera were associated with space-time cells in the modeling grid according to their geographic shapefiles and date ranges. In the space dimension, observations were geographically linked to all 20-km × 20-km grid cells that intersected the observation’s geographic shapefile. When grid cells were only partially covered by the observation’s geographic shapefile (for example, grid cells at country borders), we computed and assigned a spatial fraction (sfrac) to the grid cell–shapefile pairs. The sfrac value was calculated as the sum of the 1-km × 1-km grid cell population (after aligning the 1-km × 1-km WorldPop gridded population estimates to the 20-km × 20-km spatial modeling grid) that intersected the observation’s geographic shapefile divided by the total 20-km × 20-km grid cell population. In the time dimension, observations were mapped to all annual time slices that overlapped with the observation date range.

We removed cell–shapefile linkages with small overlaps in order to improve the smoothness of model estimates at shapefile borders. Spatial grid cells that overlapped with an observation’s geographic shapefile with a population-weighted spatial fraction below 0.05 were removed from being associated with the shapefile. To improve the smoothness of model estimates at country borders, we removed spatial grid cells from the space-time modeling grid if the grid cell sfrac was less than 0.3.

### Other spatial data sources

Gridded annual 1-km × 1-km population estimates were taken from the unconstrained global mosaic WorldPop population counts dataset and matched by cholera observation year (https://www.worldpop.org/). The gridded estimates were then linearly scaled such that the total country population matched the respective annual estimate from the 2022 revision of the United Nations *World Population Prospects*^51^. For map visualizations, we accessed major water bodies in Africa published by the Regional Centre for Mapping of Resources for Development and the AQUASTAT program of the Food and Agriculture Organization of the United Nations^52,53^.

### Statistical framework for modeling mean annual incidence

We developed a hierarchical Bayesian modeling framework that accounts for spatiotemporal heterogeneity in underlying suspected cholera incidence and variability and overlap in the spatial and temporal scales of case reports within and across data sources. In particular, the framework accounted for misalignment in spatial and temporal resolutions both across data sources and between observed case counts and intended outputs. This model expanded on a previously published approach^4^.

#### Model overview and inference

We modeled each country and time period (2011–2015 and 2016–2020) separately. The process model for most country-periods consisted of a log-linear model of annual cholera incidence rates over a 20-km × 20-km grid accounting for spatial autocorrelation and interannual variability. Spatial autocorrelation was implemented through a directed acyclic graph autoregressive (DAGAR) prior for the spatial random effects, which has improved performance relative to traditional spatial priors in disease mapping^54^. Temporal variability was modeled through annual temporal random effects. Yearly observations and temporally censored observations contributed to separate parts of the likelihood. The process models for country-periods with no or minimal subnational data were modified to improve interpretability of results and model performance (see Supplementary Table 4 for model settings by country and Methods, ‘Mean annual incidence model equations’ for model details). The modeled mean incidence rate for an area corresponding to a reported case count was derived as the weighted average of the incidence rates of grid cells covering that area. Observations overlapping in space and time were treated as independent measurements of the same underlying incidence rate. To account for reporting variability across data sources, we used a negative-binomial observation model for the case counts with inferred administrative level-specific overdispersion parameters (Methods, ‘Mean annual incidence model equations’).

Posterior samples were drawn with Hamiltonian Monte Carlo (HMC) as implemented in the Stan programming language^55^. Sampler convergence was assessed visually through the inspection of trace plots and observation-level Rhat statistics^56^. Model fit was evaluated through scatterplots of true and fitted observations and posterior retrodictive checks of the posterior coverage of observations by administrative level^57^ (Supplementary Figs. 6–15). Gridded outputs were postprocessed for mean annual incidence, mean annual incidence rate, IRR, population in ADM2 units in 5-year and 10-year incidence categories, assignment of ADM2 units in 5-year and 10-year incidence categories at ADM0 and ADM2 (sometimes called country-level and district-level, respectively) and region and continent scales for analysis (Methods, ‘Incidence modeling postprocessing’).

#### Mean annual incidence model equations

We first describe the base statistical model and add complexity that improves the base model’s ability to handle challenges presented by the real-world observation data. The core of the modeling framework consists in partitioning the variability of suspected cholera case reports among interannual variability, captured by yearly random effects, spatial patterns captured by a spatial autocorrelation prior and assumed to be constant for each 5-year modeling period and observational variability captured through an overdispersed observation model (negative binomial). Sharing spatial information across years helped improve the quality of the spatially resolved estimates given the limited availability of subnational data in many countries. We think that this partitioning of variability between temporal and spatial effects and observational overdispersion into two modeling periods represents an appropriate compromise of reliable volumes of input data, model complexity, spatial smoothing and data-driven inference to meet our analysis aim.

At the end, we present the full final ‘standard’ model structure and deviations from this standard model structure, which were deployed in country-periods described in Supplementary Table 4.

##### Base model

To estimate mean annual incidence across a period of *T* years (*T* annual time slices), we first must model annual cholera incidence estimates corresponding to a ‘modeling time resolution’ of 1 year. In a simple scenario, suppose that all observations have a duration of 1 year, which means that the ‘observation time resolution’ always equals the modeling time resolution. To model space-time incidence rates over a spatial domain that covers the area of interest across the *T* years, we defined a modeling space-time grid with a time resolution of 1 year for a given gridded spatial resolution—that is, each space-time grid-cell (*s*,*t*) spans 1 year *t* and a spatial grid cell *s*.

Observation-level cases can then be modeled as:

$${c}_{i}={\sum}_{{S}_{i,s,t}}\quad{\lambda}_{s,t}{\phi }_{i,s}{\rm{pop}}_{s,t},$$

$$\log \left({\lambda }_{s,t}\right)=\gamma +{\omega }_{s}+{\eta }_{t},$$

where ${c}_{i}$ represents the modeled mean number of cases for observation *i*; ${S}_{i,s,t}$ is the set of space-time grid cells intersecting observation *i*; ${\lambda }_{s,t}$ is the annual incidence rate in space-time grid cell *s*,*t*; ${\phi }_{i,s}$ is the population-weighted spatial fraction of grid cell *s*,*t* that is covered by the observation location; and ${\rm{pop}}_{s,t}$ is the total population in grid cell *s*,*t*. Grid cell incidence rates were modeled with a log link as the sum of the offset $\gamma$, which is the expected incidence rate across the space-time modeling grid, spatial random effect ${\omega }_{s}$ and yearly random effect ${\eta }_{t}$.

The expected incidence rate $\gamma$ was calculated as the population-weighted mean of the implied incidence rate (time-adjusted reported cases of full-year observations divided by location population) across all full-year observations contributing to the model (see Methods, ‘Identifying time-censored observations’). If the expected incidence rate was less than 0.01 per 100,000 population, it was changed to be 1 × 10^−7^.

Observation ${y}_{i}$ is then linked to modeled cases through an observation model. For instance, in the simplest setting, one can assume that observations follow a Poisson distribution:

$${y}_{i}\sim {\rm{Poisson}}({c}_{i}).$$

However, a Poisson model does not reflect the heterogeneity in case counts observed in the data, thereby necessitating a more elaborate observation process model. We expand on the observation process below.

##### Prior on the spatial random effect (*ω*_*s*_)

To capture spatial variability in the incidence rates and produce spatially smooth maps, we introduced spatial random effects into the model at the grid cell level. We assumed that the spatial random effect ${\omega }_{s}$ was constant across the *T* time slices to reduce the number of parameters that must be estimated from a model that may have limited observations in any given time slice. We acknowledge that this model may not adequately capture situations where the spatial autocorrelation in cholera cases changes across time slices. In such scenarios, the estimates of ${\omega }_{s}$ will represent the average spatial variability across all the time slices.

In our model, the joint distribution of ${\omega }_{s}$ for all spatial grid cells *s* is specified as a DAGAR prior. The DAGAR model was demonstrated to have improved model performance, interpretability of parameters and computational efficiency over other spatially smooth priors that are traditionally used in disease mapping (for example, conditional autoregressive prior)^54^. The DAGAR prior can be specified via a sequence of simple conditional normal distributions. Specifically, the conditional distribution of ${\omega }_{s}$, conditional on its directed neighbors on the grid, follows a normal distribution with mean ${\mu }_{{\omega }_{s}}$ and s.d. ${\sigma }_{{\omega }_{s}}$:

$${\omega }_{s}\sim {\rm{Normal}}\left({\mu }_{{\omega }_{s}},{\sigma }_{{\omega }_{s}}\right),$$

$${\mu }_{{\omega }_{s}}=\frac{\rho }{(1+(n{n}_{s}-1){\rho }^{2})}\sum _{u\in {\varOmega }_{s}}{\omega }_{u},$$

$${\sigma }_{{\omega }_{s}}={\xi }_{{\sigma }_{w}}\sqrt{\frac{(1-{\rho }^{2})}{(1+(n{n}_{s}-1){\rho }^{2})}},$$

where $\rho$ is the strength of the spatial autocorrelation between grid cells; ${{nn}}_{s}$ is the number of neighbors of cell *s*; and ${\varOmega }_{s}$ is the set of neighbors to cell *s*. We denote the DAGAR prior as ${\mathbf{\upomega }} \sim {\rm{DAGAR}}(\rho ,{\xi }_{{\sigma }_{w}})$ where ${\mathbf{\upomega}}$ is a vector containing ${\omega }_{s}$ for all the spatial grid cells *s*.

##### Prior on the temporal random effects (${\eta }_{t}$)

Although the yearly temporal random effects were initially assumed to be independent, we imposed a zero-sum constraint to improve identifiability of these parameters and enforced a marginal standard normal prior on the set of these terms^58^. In brief, the approach applies a QR decomposition on the covariance matrix of the yearly random effect to obtain a set of random variables with a zero-sum constraint and marginal s.d. values of 1. In practice, priors are set on *T* *−* 1 independent random effects, and the random effect of the *T-*th time slice is computed from them.

##### Expansion for partial-year observations

Partial-year observations (that is, those with tfrac < 0.65 within a given annual modeling time slice) were treated as right-censored in the likelihood (see Methods, ‘Identifying time-censored observations’). Because we assumed that incidence rates were non-homogeneous within a given annual time slice, we chose to treat partial-year observations as right-censored observations of the annual counts as opposed to performing an extrapolation to represent a full year. In other words, we make no assumptions beyond that the number of cases in the full-year modeling time slice would be at least as large as the number of observed cases in the partial-year observation. The observation model likelihood for partial-year observations was:

$$L(\,{y}_{i})=\Pr (Y\ge {y}_{i}|{c}_{i}),$$

So, in the case of the Poisson observation model, the likelihood is:

$$L\left(\,{y}_{i}\right)=1-{\rm{CDF}}_{\rm{Poisson}}\left(\,{y}_{i}|{c}_{i}\right).$$

##### Expansion for overdispersed observation data

Examination of the observation data determined that a Poisson observation model would not be sufficient to account for the overdispersion observed in many country-period models and across different administrative unit levels. Consequently, we accounted for overdispersion with a negative binomial observation likelihood:

$${y}_{i}\sim {\rm{NegBinom}}\left({c}_{i}, {\tau }_{A\left[i\right]}\right),$$

where *τ* is the overdispersion parameter that defines the relationship of the mean $c$ to the variance:

$${\rm{variance}}=c+\frac{{c}^{2}}{\tau }.$$

To account for expected differences in overdispersion across administrative level reporting, the model allowed for different overdispersion parameters by observation administrative unit level *A[i]*. The overdispersion parameter (${\tau }_{A0}$) was fixed at the country level (A0) but inferred for all other administrative unit levels.

##### Complete standard model formulation

The final standard model followed a hierarchical structure, such that the process model was defined:

$${c}_{i}=\sum _{{S}_{i,s,t}}\quad{\lambda }_{s,t}{\phi }_{i,s}{po}{p}_{s,t},$$

$$\log \left({\lambda }_{s,t}\right)=\gamma +{\omega }_{s}+{\eta }_{t},$$

and the observation model was defined for full-year and partial-year observations:

$$\Pr(\,{y}_{i}|{c}_{i})={\rm{NegBinom}}(y_i|{c}_{i}, {\tau }_{A[i]}) \qquad \text{if} \quad {\varPhi}_{i,t}\ge a,$$

$$\Pr \left(\,{y}_{i}|{c}_{i}\right)=1-{\rm{CDF}}_{\rm{NegBinom}}\left({y}_{i}|{c}_{i},{\tau }_{A\left[i\right]}\right)\qquad \text{if} \quad {\varPhi }_{i,t} < a,$$

where $a=0.65$, the threshold above which the time fraction ${\varPhi }_{i,t}$ for observation *i* is considered to represent the full year *t*.

We used the following hyperpriors for the spatial random effects:

$${\mathbf{\upomega }} \sim {\rm{DAGAR}}\left(\rho ,{\xi }_{{\sigma }_{w}}\right),$$

$$\rho \sim {\rm{Beta}}\left(5,1.5\right),$$

$$\Pr \left({\xi }_{{\sigma }_{w}}\right)=\theta {f}_{\rm{Normal}}\left({\xi }_{{\sigma }_{w}}|10,{\sigma }_{\omega ,1}^{{\prime} }\right)+\left(1-\theta \right){f}_{\rm{Normal}}\left({\xi }_{{\sigma }_{w}}|0,{\sigma }_{\omega ,2}^{{\prime} }\right),$$

$$\theta \sim {\rm{Beta}}\left(1,3\right),$$

$${\sigma }_{\omega ,1}^{{\prime} }\sim {\rm{Half}\; \rm{normal}}\left(0,2\right),$$

$${\sigma }_{\omega ,2}^{{\prime} }\sim {\rm{Half}\; \rm{normal}}\left(0,0.5\right),$$

where $\Pr ({\xi }_{{\sigma }_{w}})$ represents a mixture prior on the s.d. scaling constant (${\xi }_{{\sigma }_{w}}$), which is the sum of two normal distribution densities (${f}_{\rm{Normal}}$), one centered at 0 and the other centered at 10, weighted by the mixture parameter $(\theta )$. The hyperpriors on the s.d. of the normal distribution densities contributing to the mixture prior are represented by ${\sigma }_{\omega ,1}^{{\prime} }$ and ${\sigma }_{\omega ,2}^{{\prime} }$. This mixture prior reflects the possibility that different models may have high or low magnitude of spatial variability.

We used the following prior for temporal random effects:

$${\eta {\prime} }_{[1:T-1]}\sim {\rm{Normal}}\left(0,\frac{1}{\sqrt{1-\frac{1}{T}}}\right),$$

where ${\eta {\prime} }_{[1:T-1]}$ is multiplied by the QR matrix (see Methods, ‘Prior on the temporal random effects’) to yield the ${\eta }_{[1:T]}$ yearly random effects for all *T* time slices whose sum is enforced to be 1.

For the observation model, the overdispersion term for administrative unit level 0 (*A0*) observations was fixed at 100 or 1,000, which corresponded to a moderate amount of overdispersion for case counts of that magnitude. We used the following priors for the overdispersion term in the observation model:

$${\tau }_{A0}=100\quad{{\rm{if}}\; {\rm{max}}}(\,{y}_{i,A0})\le 5{,}000,$$

$${\tau}_{A0}=1{,}000\quad{{\rm{if}}\;{\rm{max}}}(\,{y}_{i,A0})>5{,}000,$$

$$\frac{1}{{\tau }_{A > 0}}\sim {{\rm{Half}}\; {\rm{normal}}}\left(0,1\right),$$

where *A* > 0 refers to administrative level units below the national level.

##### Model formulation without spatial autoregressive term

For country-periods with no subnational observations or only zero-case observations, we removed the spatial random effect from the process model. For this model deviation, spatial random effect priors were removed, and the process model was as follows:

$${c}_{i}={\sum}_{{S}_{i,s,t}}\quad{\lambda}_{s,t}{\phi}_{i,s}{\rm{pop}}_{s,t},$$

$$\log \left({\lambda }_{s,t}\right)=\gamma +{\eta }_{t}.$$

##### Model selection and model formulation with non-mixture prior

All country-periods with at least one subnational non-zero-case observation were first attempted with the standard model formulation. We found that models with limited subnational data had poor model fit and convergence due to identifiability issues in the spatial autocorrelation strength parameter $\rho$ determining the spatial random effect $\omega$. The standard model employed a mixture prior on the scaling factor (${\xi }_{{\sigma }_{w}}$) of the s.d. of $\omega$ (${\sigma }_{{\omega }_{s}}$) to account for possible bimodality in the strength of spatial autocorrelation (that is, country-periods may have high or low $\rho$ depending on the data).

For country-periods with limited subnational data, model convergence improved when the mixture prior on ${\xi }_{{\sigma }_{w}}$ was replaced with a unimodal prior that favors a higher ${\sigma }_{{\omega }_{s}}$ and, therefore, lower $\rho$. The scaling constant priors for this model deviation reduced to:

$${\xi }_{{\sigma }_{w}}\sim {{\rm{Half}}\; {\rm{normal}}}(5,0.5).$$

#### Incidence modeling postprocessing

Our modeling framework produced 20-km × 20-km gridded mean annual incidence estimates for each country and time period (4,000 posterior samples). Grid cell estimates were then aggregated to ADM0 (country) and ADM2 (district) scales according to standardized sets of non-overlapping country-unified shapefiles. These were obtained for each modeled country from GADM (https://gadm.org/) or geoBoundaries^49^ after an additional quality assessment (Supplementary Table 5). Lesotho did not have ADM2 shapefiles from a standard source, so these outputs were processed to the ADM1 scale instead.

A set of continent-wide and region-wide posterior samples was assembled by summing across a single random posterior predictive sample of each country model; mean and 95% CrIs were then calculated from the summed results, thus yielding 4,000 continent-wide and region-wide posterior predictive samples that are internally consistent.

Multiple outputs were calculated at continent, regional, ADM0 and ADM2 scales for each model in postprocessing: mean annual incidence (cases per year); mean annual incidence rate (cases per population per year); IRR (calculated as mean annual incidence rate in 2016–2020 divided by that in 2011–2015); assignment of ADM2 units to 5-year and 10-year incidence categories; and population in ADM2 units in 5-year and 10-year incidence categories. Mean annual incidence and mean annual incidence rate posterior mean and 95% CrI estimates were calculated across 4,000 posterior predictive samples at the relevant spatial scale. To estimate IRRs, the mean and 95% CrIs were calculated across all pairwise ratios of 4,000 posterior predictive samples in each period (that is, evaluated across the distribution of 4,000^2^ samples—all 2016–2020 samples were pairwise-divided by all 2011–2015 samples). IRRs were deemed statistically significant if the 95% CrIs did not cross 1.

The number of people living in ADM2 units by 5-year incidence categories at continent and region scales was estimated across the summed 4,000 continent-wide and region-wide posterior samples described above (corresponds to results in Fig. 3a and Extended Data Fig. 5). The mean and 95% CrIs for each 5-year incidence category were calculated across the 4,000 posterior samples. Consequently, the variability in these estimates reflects variation in ADM2 incidence category assignment across samples.

Assignment of ADM2 units to specific 5-year incidence categories was performed in a two-step procedure (corresponds to results in Fig. 3b and Extended Data Fig. 6). First, we determined incidence categories for each 20-km × 20-km modeling grid cell and posterior sample and retained the highest incidence category encompassing at least 10% of the unit’s population or 100,000 people in 2020. Then, ADM2 units were assigned to the lowest incidence category with at least 50% posterior cumulative probability of assignment at or above that level (for example, ADM2 unit was assigned to the 50–100 cases per 100,000 category if ≥50% of posterior samples categorized the ADM2 unit in the 50–100 or ≥100 cases per 100,000 people categories). Thus, the assignment of an ADM2 unit to an incidence category already factors in the variability in assignment across samples. We note that summing the number of people in all ADM2 units by 5-year incidence category would not yield the same number as the continent-wide mean estimate of population living in the ADM2 category.

Assignment of ADM2 units to 10-year incidence categories was based directly on their 2011–2015 and 2016–2020 incidence category assignments. We defined four 10-year incidence categories: ‘sustained high’ for ADM2 units classified as high incidence (≥10 cases per 100,000 people per year) in both periods; ‘history of high’ for ADM2 units classified as high incidence in at least one period; ‘sustained low’ for ADM2 units classified as <1 case per 100,000 per year in both periods; and ‘history of moderate’ for all other combinations.

#### Consideration of border effects between countries

We ran our statistical model on each country separately, which could lead to border effects in our mean annual incidence estimates between countries. Independent country models sometimes generated estimates for the same border grid cell, in which case we merged the estimates taking the mean across posterior samples from the model runs of each neighboring country. During model validation, we performed a close examination of border estimates out of concern that country-level modeling may introduce artificial edge effects and did not observe outlier or unusual estimates at country borders (Fig. 1b).

Although we did not explicitly model cross-border transmission, cross-border regions of higher cholera incidence were able to be captured directly from subnational model input data (Extended Data Figs. 1 and 2). For example, the modeled estimates appear to identify contiguous high-incidence areas across several country borders, including Nigeria–Cameroon–Chad, South Sudan–Kenya and DRC–Zambia. We, however, note that some cross-country borders had notable discontinuities in mean annual incidence estimates (for example, the Southern Malawi–Northern Mozambique border). These could be due to real differences in underlying cholera incidence or due to differences in cholera reporting between administrative areas. As such, these discontinuities are valuable opportunities for further investigations of the spatial patterns of cholera incidence and reporting in Africa, which we intend to exploit in future work.

### Definition of 5-year and 10-year incidence categories

We identified six categories pertaining to the 5-year mean annual incidence: <1, [1, 10), [10, 20), [20, 50), [50, 100) and ≥100 cholera cases per 100,000 people per year. For a given posterior sample, ADM2 units were assigned to the most severe 5-year incidence category where at least 10% of the unit’s population or 100,000 people (in 2020-adjusted population sizes) were living, according to the modeled 20-km × 20-km grid cell estimates (see Methods, ‘Incidence modeling postprocessing’ for details). This incidence category assignment procedure was designed to identify locations that may make high-impact targets for public health intervention. We used the labels ‘very high incidence’ to refer to the category of ≥100 cases per 100,000 population per year, ‘high incidence’ for ≥10 per 100,000 and ‘low incidence’ for <1 per 100,000.

We also used a 10-year incidence categorization at the ADM2 level that combines the incidence categories in each 5-year period (2011–2015 and 2016–2020). We defined four 10-year incidence categories: ‘sustained high’ for ADM2 units classified as high incidence (≥10 cases per 100,000 people per year) in both periods; ‘history of high’ for ADM2 units classified as high incidence in at least one period; ‘sustained low’ for ADM2 units classified as <1 case per 100,000 per year in both periods; and ‘history of moderate’ for all other combinations.

People living in 5-year incidence categories relied on the mean population estimate across the relevant 5-year period. We used 2020 population estimates for people living in 10-year incidence categories and the analysis of potential intervention reach in order to facilitate comparison of epidemiologic changes over time.

### Statistical analysis of 2022–2023 cholera occurrence

We evaluated the association between the 10-year incidence categories (from 2011 to 2020) and cholera occurrence as reported in 2022–2023 WHO external cholera situation reports 1 through 10 (refs. ^1,59–67^), complemented with country-specific situation reports for areas not displayed in WHO reports^68–72^ (Supplementary Table 6). The extracted data corresponded primarily to cholera reported between January 2022 and December 2023, with limited additional data in surrounding months due to the temporal reporting resolution. The 2022–2023 period was selected for analysis because it corresponded to the WHO emergency declaration period, and centralized and official WHO datasets were available for data extraction. For this analysis, locations were determined to have cholera if one or more suspected cholera cases were reported in any of the above-described situation reports.

Fourteen situation report documents (Supplementary Table 6) with map images of cholera occurrence in the post-2020 period were loaded into QGIS geographic information system software (version 3.28.12) and overlaid with the standardized set of country-unified shapefiles (Supplementary Table 5). Each image was georeferenced to the country-unified shapefiles as a basemap with country borders as control points. After aligning the administrative unit boundaries, we manually added centroids to extract point locations for each administrative unit and added attributes to identify the administrative unit level, confidence about the certainty of the administrative unit level, presence of reported cholera cases and the time range represented by the map.

We then spatially joined extracted locations with cholera occurrence to the set of unique ADM2 units used to summarize the cholera incidence mapping results. Cholera occurrence was extrapolated to ADM2 units if cholera was reported in ADM3 scale units or below.

#### Occurrence model equations

As the situation reports provided limited subnational case data, we evaluated the association between 10-year incidence categories and recent cholera occurrence (binary outcome) in a Bayesian modeling framework. The model consisted of a hierarchical logistic regression that accounted for the probability of failing to detect cholera (false negatives), reporting of cases at multiple administrative levels as well as partial pooling of country-specific parameter values at the regional and continental levels. Inference was performed with HMC as implemented in the Stan programming language^55^.

We first describe a base statistical model and add hierarchical spatial complexity to complete the full model description.

##### Base statistical model

This analysis aimed to estimate the association between 10-year incidence categories and the probability of reporting suspected cholera occurrence in the 2022–2023 period. For all locations that were modeled, those that reported cholera were indexed with *j*, and those that did not report cholera were indexed with *k*.

For ADM2 locations that reported cholera occurrence, the likelihood is:

$$L(\,{y}_{\!j,A2}=1)={p}_{\!j,A2}{\phi }_{j,A2},$$

and

$${\rm{logit}}\left({p}_{\!j,A2}\right)=\alpha +{\beta }_{j,A2},$$

where ${y}_{\!j,A2}$ is the reported cholera occurrence status extracted from the situation report documents in location *j*, which is at the ADM2 level ($A2$); ${p}_{\!j,A2}$ is the probability of true cholera occurrence; ${\phi }_{j,A2}$ is the probability of reporting cholera if it is present (sensitivity of cholera detection); $\alpha$ is the model intercept; and ${\beta }_{j,A2}$ is the effect of the 10-year incidence category in the ADM2 unit. Notably, the model assumes that all reported cholera is a true instance of cholera occurrence (that is, no false positives).

As the absence of reported occurrence may be due to lack of cholera occurrence or lack of reporting, we treated the absence of reported occurrence as missing data and marginalized out all possible reporting statuses to estimate the underlying true cholera occurrence status. For ADM2 locations that did not report cholera, the likelihood reads:

$$L(\,{y}_{k,A2}=0)={p}_{k,A2}(1-{\phi}_{k,A2})+(1-{p}_{k,A2})=1-{p}_{k,A2}{\phi}_{k,A2},$$

and

$${\rm{logit}}\left({p}_{k,A2}\right)=\alpha +{\beta }_{k,A2},$$

where ${p}_{k,A2}$ is the probability of true cholera occurrence in ADM2 unit $k$; $(1-{\phi }_{k,A2})$ is the probability of not reporting cholera if it is indeed present; and ${\beta }_{k,A2}$ is the effect of the 10-year incidence category in the ADM2 unit.

Reports of cholera occurrence in ADM2 locations could, therefore, be modeled with a Bernoulli distribution:

$${y}_{i,A2}\sim {\rm{Bernoulli}}\left({p}_{i,A2}{\phi }_{i,A2}\right),$$

where *i* represents any location regardless of cholera reporting status.

##### Adding higher administrative unit level observations

As some occurrence data were available only at the ADM1 or ADM0 (country) level, these observations were integrated into the model:

$${y}_{i,A < 2}\sim {\rm{Bernoulli}}({\eta }_{i,A < 2}),$$

and

$${\eta }_{i,A < 2}=1-\prod _{i,A2\in S,A2}1-{p}_{i,A2}{\phi }_{i,A2},$$

where *S*,*A2* represents the set of *i*,*A2* ADM2 units contained within the location *i*,*A* < *2*, which is above the ADM2 level, and ${\eta }_{i,A < 2}$ is the probability of reported cholera occurrence in the higher administrative unit level location *i*,*A* < *2*.

##### Hierarchical country-level and region-level priors

We assumed that the association between 10-year incidence categories and the probability of cholera occurrence may vary across countries and regions (for example, eastern Africa). We accounted for these geographic differences by setting hierarchical priors, such that priors for the association of the 10-year incidence category and probability of true cholera occurrence in location *i*, which is contained within country $c$ and region $r$, were defined as:

$${\beta}_{c}^{m}{\sim}{\rm{Normal}}\left(\,{\mu}_{\beta ,r}^{m},{\sigma}_{\beta,r}^{m}\right),$$

and

$${\mu }_{\beta ,r}^{m}\sim {\rm{Normal}}\left({\mu }_{\beta }^{m},{\sigma }_{\beta }^{m}\right),$$

where $m$ denotes the 10-year incidence category associated with location *i*; ${\mu }_{\beta ,r}^{m}$ and ${\sigma }_{\beta ,r}^{m}$ are regional-level mean and s.d. of the 10-year incidence category effect $\beta$; and ${\mu }_{\beta }^{m}$ and ${\sigma }_{\beta }^{m}$ are hyperpriors for the mean and s.d. of the 10-year incidence category effect.

Hierarchical priors were also assumed for the cholera detection sensitivity parameters $\phi$, which had analogous relationships on a logit scale:

$${\rm{logit}}({\phi}_{c}){\sim}{\rm{Normal}}(\,{\mu}_{{\rm{logit}}(\phi),r},{\sigma}_{{\rm{logit}}(\phi),r}),$$

and

$${\mu }_{{\rm{logit}}(\phi ),r}\sim {\rm{Normal}}\left({\mu }_{{\rm{logit}}\left(\phi \right)},{\sigma }_{{\rm{logit}}\left(\phi \right)}\right).$$

##### Model priors and hyperpriors

We used the following priors:

$${\sigma }_{\beta ,r}^{m}\sim {{\rm{Half}}\; {\rm{normal}}}\left(0,2.5\right),$$

$${\mu }_{\beta }^{m}\sim {\rm{Normal}}\left(0,2\right),$$

$${\sigma }_{\beta }^{m}\sim {{\rm{Half}}\; {\rm{normal}}}\left(0,2.5\right),$$

$${\sigma }_{{\rm{logit}}(\phi ),r}\sim {{\rm{Half}}\; {\rm{normal}}}\left(0,1\right),$$

$${\mu }_{{\rm{logit}}(\phi )}\sim {\rm{Normal}}\left(1.5,5\right),$$

$${\sigma }_{{\rm{logit}}(\phi )}\sim {{\rm{Half}}\; {\rm{normal}}}\left(0,1\right).$$

### Assessing potential intervention reach when prioritizing targets by cholera incidence

We assessed the potential reach (best-case scenario) of non-specific interventions when targeted based on cholera incidence through two analyses. Both analyses prioritized ADM2 units by decreasing incidence category and decreasing population size within incidence categories as a simplification of how intervention targets might be prioritized using cholera incidence data. Here, the population targeted by interventions was calculated as the sum of the targeted ADM2 unit populations, adjusted for 2020 population size. The analyses then examined what proportion of (1) mean annual cholera cases or (2) population living in ADM2 units with 2022–2023 cholera occurrence would have been reached under different targeting strategies. ‘Prospective’ targeting used past incidence categories to target future interventions, whereas ‘oracle’ targeting prioritized interventions based on incidence categories from the same period.

In the first analysis, we assessed the proportion of mean annual 2016–2020 cholera cases that would have been reached by interventions had 2011–2015 (‘prospective’) or 2016–2020 (‘oracle’) incidence categories been used for targeting. We also assessed the proportion of mean annual 2011–2015 cholera cases that would have been reached by interventions had 2011–2015 incidence categories been used for targeting (‘oracle’ only).

In the second analysis, we examined the proportion of population living in ADM2 units with modeled 2022–2023 cholera occurrence (modeled according to the above-described statistical analysis) that would have been reached by interventions had 2011–2015, 2016–2020 and 2011–2020 incidence categories (‘prospective’) been used for targeting. These three strategies were compared to an ‘oracle’ targeting strategy where ADM2 units with 2022–2023 cholera occurrence were ranked in decreasing order of population size.

### Inclusion and ethics statement

The institutional review board (IRB) at Johns Hopkins Bloomberg School of Public Health (BSPH) determined that secondary analysis of data from the global cholera incidence database was exempt (BSPH IRB no. 27682), and no other institutional approvals were sought.

Twelve authors (J.P.M.L., R.C., P.W.O., G.B., L.E., A.V.N., N.F.M., E.W.O., S.Y., F.K., S.O.O. and A.J.S.) contribute to public health activities in cholera-affected LMICs. They provided feedback on the interpretation and application of this work based on their expertise in cholera surveillance and control in LMIC contexts. We fully endorse the Nature Portfolio journals’ guidance on LMIC authorship and inclusion and are strongly committed to the inclusion of more researchers and decisionmakers from LMICs in future related work.

Policymakers in the Africa region may use the data from this study to identify areas with historically sustained, sporadic and limited cholera activity, which may inform future cholera control planning and serve as a benchmark for measuring progress in cholera control efforts. These burden maps may also be used to identify cross-border areas that would benefit from enhanced regional coordination and surveillance.

### Reporting summary

Further information on research design is available in the Nature Portfolio Reporting Summary linked to this article.

## Online content

Any methods, additional references, Nature Portfolio reporting summaries, source data, extended data, supplementary information, acknowledgements, peer review information; details of author contributions and competing interests; and statements of data and code availability are available at 10.1038/s41591-025-03847-9.

## Supplementary information

Supplementary Information Supplementary Figs. 1–15 and Tables 1–6.

Reporting Summary

## Figures and Tables

**Extended Data Table 1:**
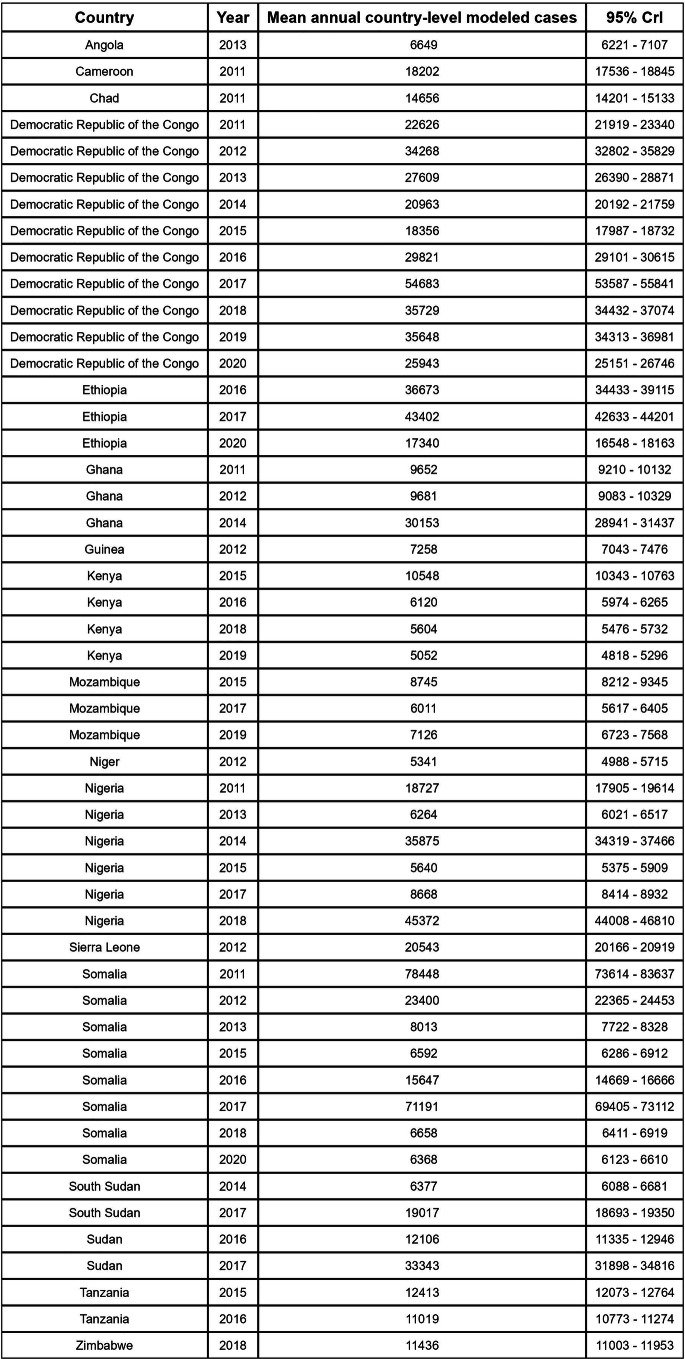
List of countries and years when the annual country-level modeled cases exceeded 5,000 cases

The table indicates the mean annual country-level modeled cases and 95% CrI across 4,000 samples from the posterior distribution.

## Data Availability

Cholera incidence datasets derived from public sources may be viewed and accessed from http://cholera-taxonomy.middle-distance.com with no restrictions while the database is maintained, at minimum for 3 years after publication. Metadata for non-public cholera incidence datasets may be requested from the corresponding author with a projected 2-week turnaround while the database is maintained and a 3-week turnaround after the database has been archived. Non-public incidence datasets will not be shared, in concordance with data-sharing agreements. Spatial population distributions were obtained from the WorldPop global unconstrained mosaic population counts product (https://www.worldpop.org), and country-level population estimates were obtained from the United Nations Population Division *World Population Prospects 2022*. Underlying maps for the Democratic Republic of the Congo, Burundi, Ethiopia, Malawi and Uganda were obtained from geoBoundaries, which has a CC BY 4.0 license. All other underlying country maps were obtained from GADM, which has a license that allows for open-access academic publishing. Gridded, ADM2, country-level and region-level modeled outputs are available on the Open Science Framework at https://osf.io/jzquw/ with no restrictions.

## Code availability

Data processing and modeling code is publicly available with a GPL v3 license on GitHub at https://github.com/HopkinsIDD/cholera-mapping-pipeline, release version 1.1. Statistical modeling and figure generation were performed on R version 4.0.3.
