# Supplementary material for: Geographical shifting of cholera burden in Africa and its implications for disease control
Source: Nat Med. 2025 Aug 7;31(10):3380–7. doi: 10.1038/s41591-025-03847-9 (PMC12532610; doi:10.1038/s41591-025-03847-9)
Supplement: Supplementary file 1 — Supplementary Figs. 1–15 and Tables 1–6. [file 41591_2025_3847_MOESM1_ESM.pdf]

nature medicine

Supplementary information https://doi.org/10.1038/s41591-025-03847-9

Geographical shifting of cholera burden in Africa and its implications for disease control

In the format provided by the authors and unedited

Supplementary Figures

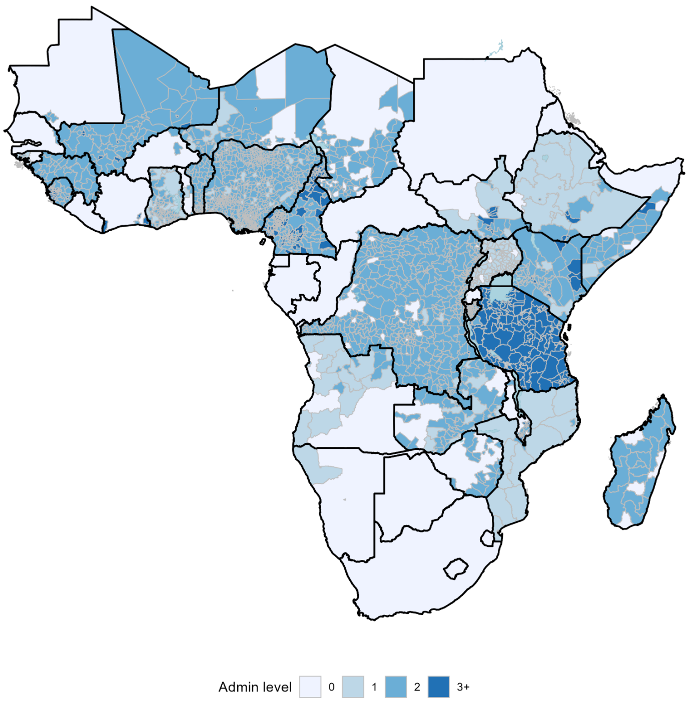

Figure S1. Spatial coverage of cholera observation data for the 2011-2015 period. Colors represent the smallest administrative unit level with at least one observation available during this period. For example, any spatial area with a pale blue fill was covered only by an administrative unit level 0 (country-level) observation, while any spatial area with a deep blue fill was covered by at least one administrative unit level 3 (subdistrict-level) observation. Only modeled countries are shown.

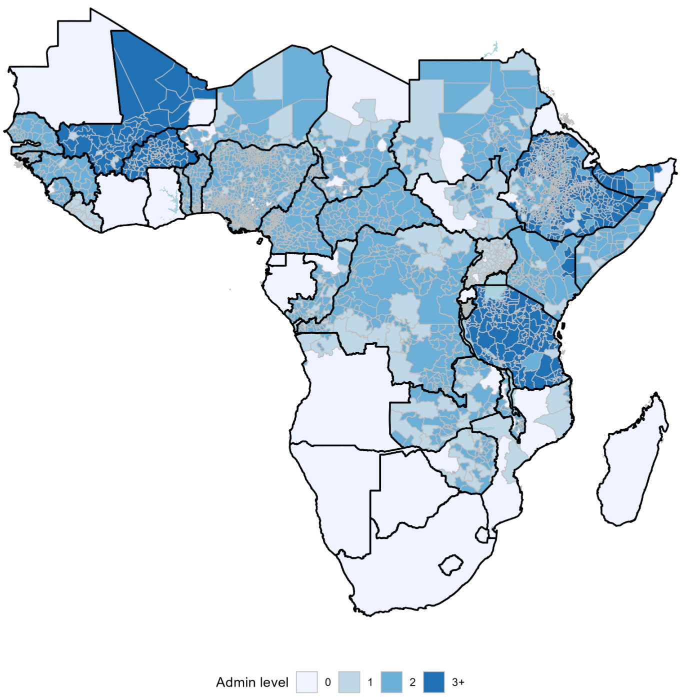

Figure S2. Spatial coverage of cholera observation data for the 2016-2020 period. Legend as in Figure S1.

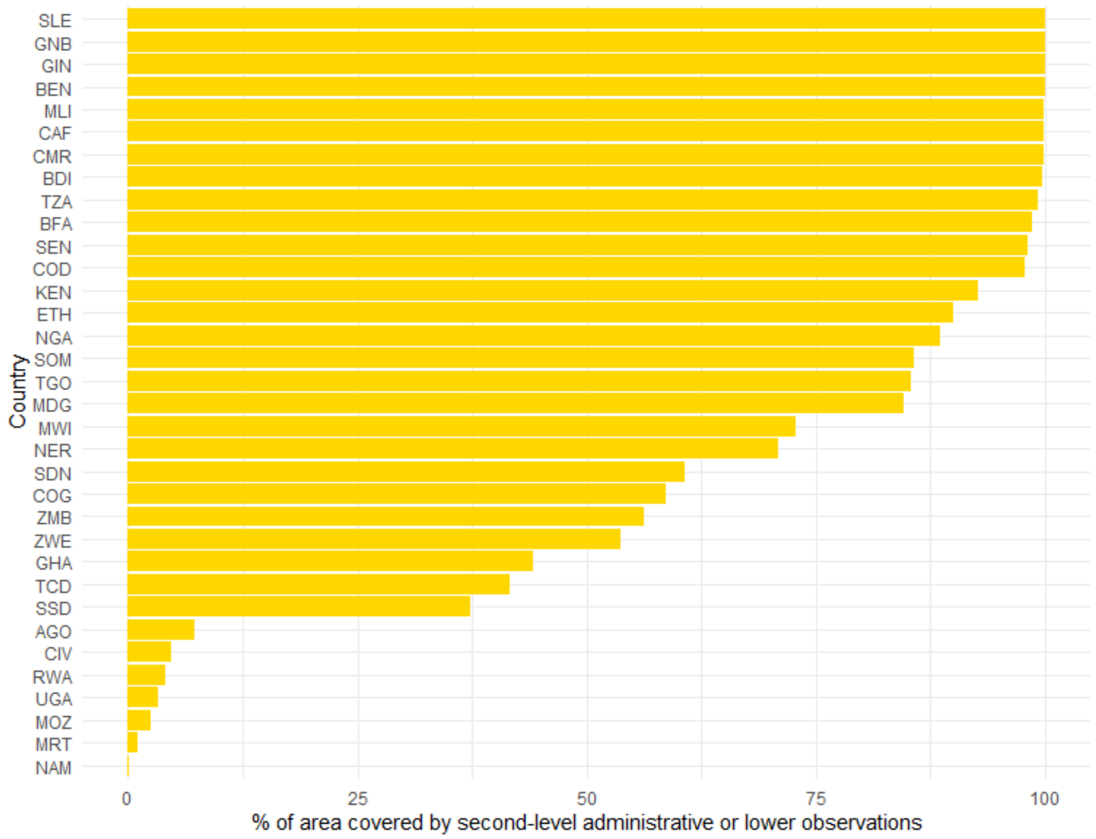

Figure S3. Percent of spatial area in the country that is covered by administrative unit level 2 or lower observations in at least one year from 2011-2020. Countries are displayed in descending rank order.

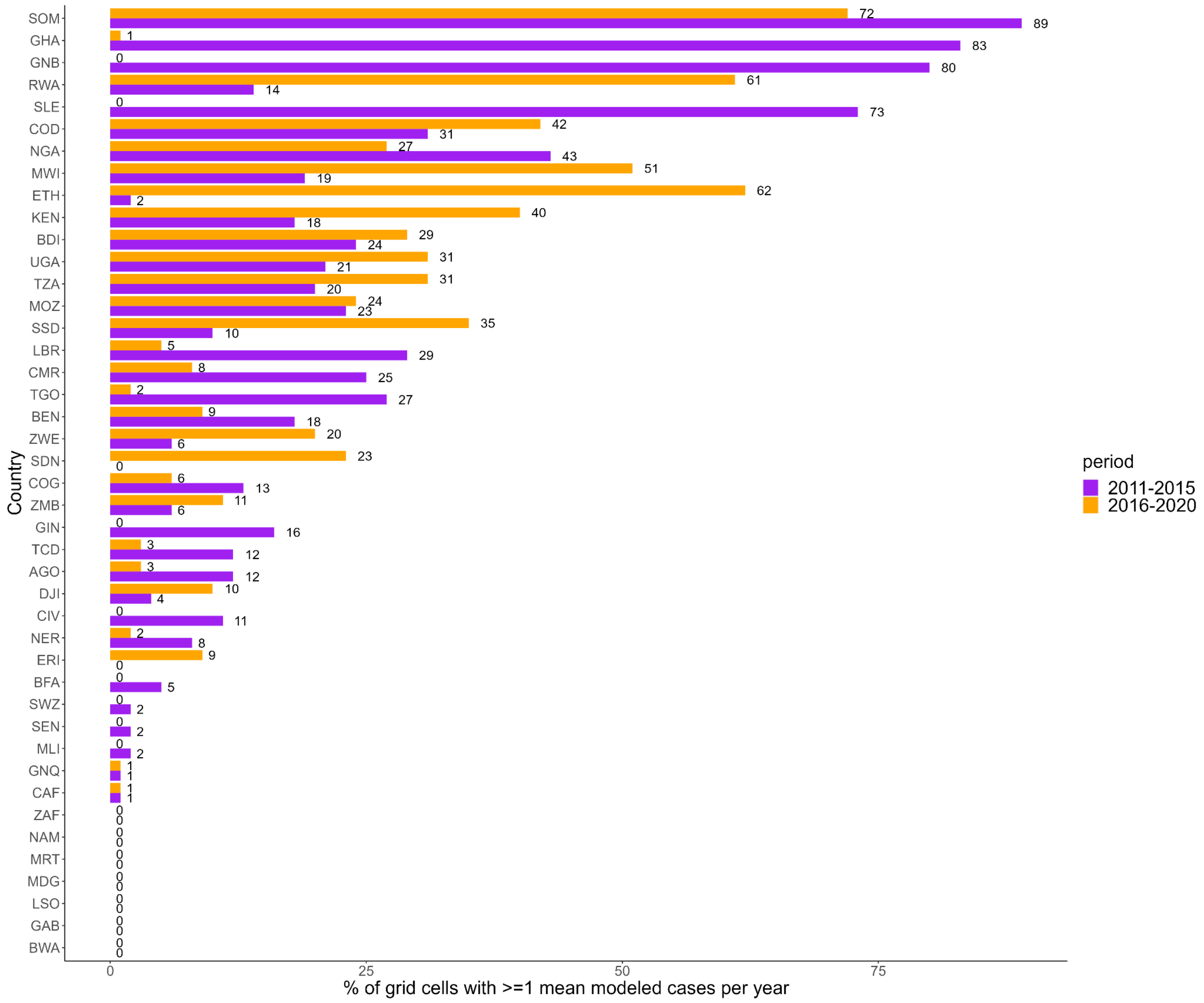

Figure S4. Percent of 20 km by 20 km grid cells in the country spatial modeling grid with at least 1 suspected case estimated per year, by country and period. Colors differentiate the two periods and labels indicate the percent value.

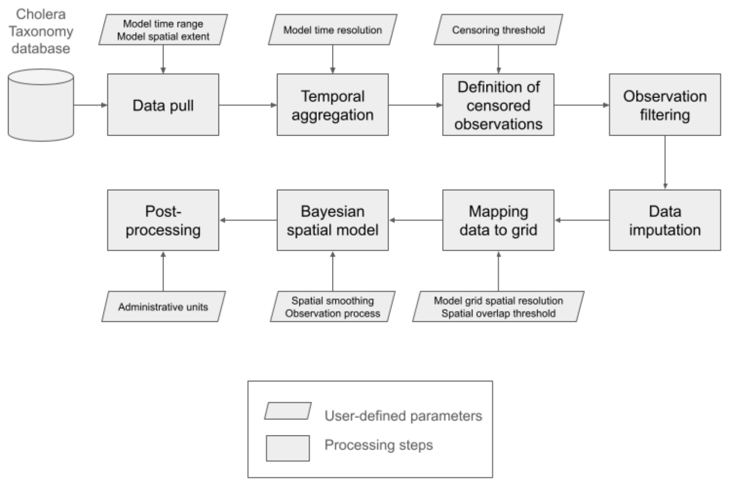

Figure S5. Flowchart summarizing the data processing and modeling pipeline and the corresponding user-defined inputs.

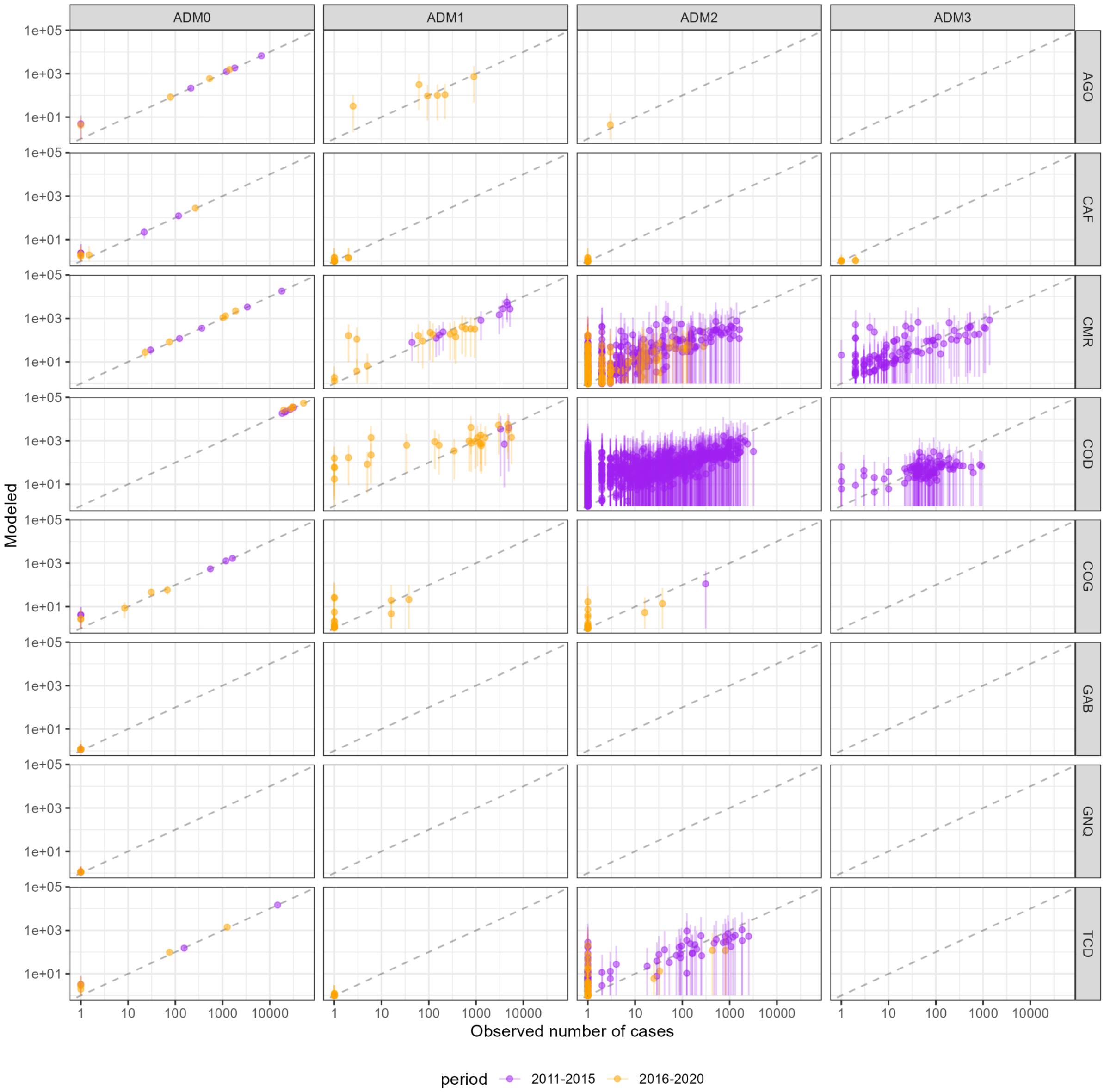

Figure S6. Scatterplot of full-year observations versus fitted values by administrative unit levels 0 to 3 for countries in Central Africa (log-scale). Dots and lines represent the mean and 95% CrI and colors indicate observations in different periods. Points falling along the 1:1 diagonal black dashed line indicates alignment between modeled and observed cases.

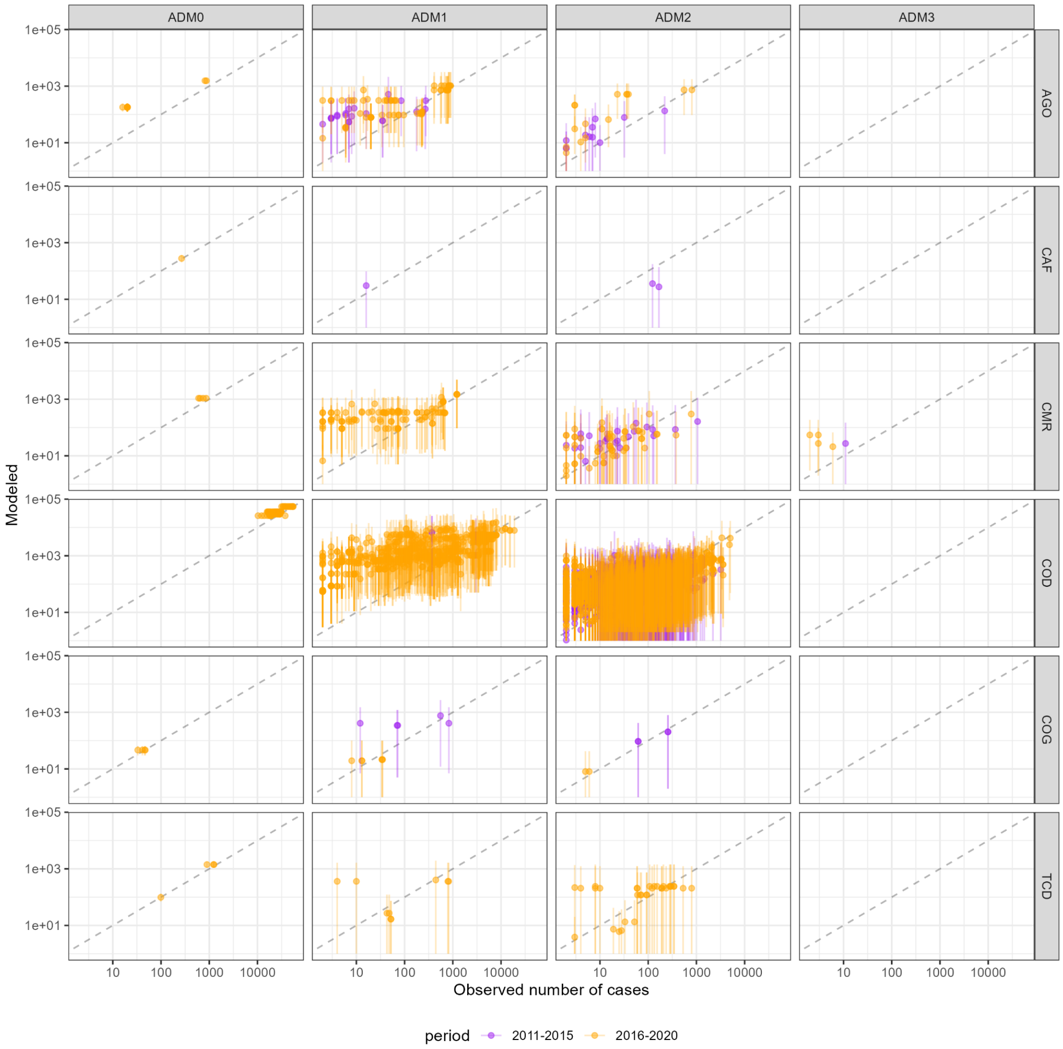

Figure S7. Scatterplot of partial-year observations versus fitted values by administrative unit levels 0 to 3 for countries in Central Africa. Dots and lines represent the mean and 95% CrI and colors indicate observations in different periods. Partial-year observations were treated as right-censored in the model likelihood. Models that fit well should have fitted values (y-axis) at or above their corresponding model observation values (x-axis).

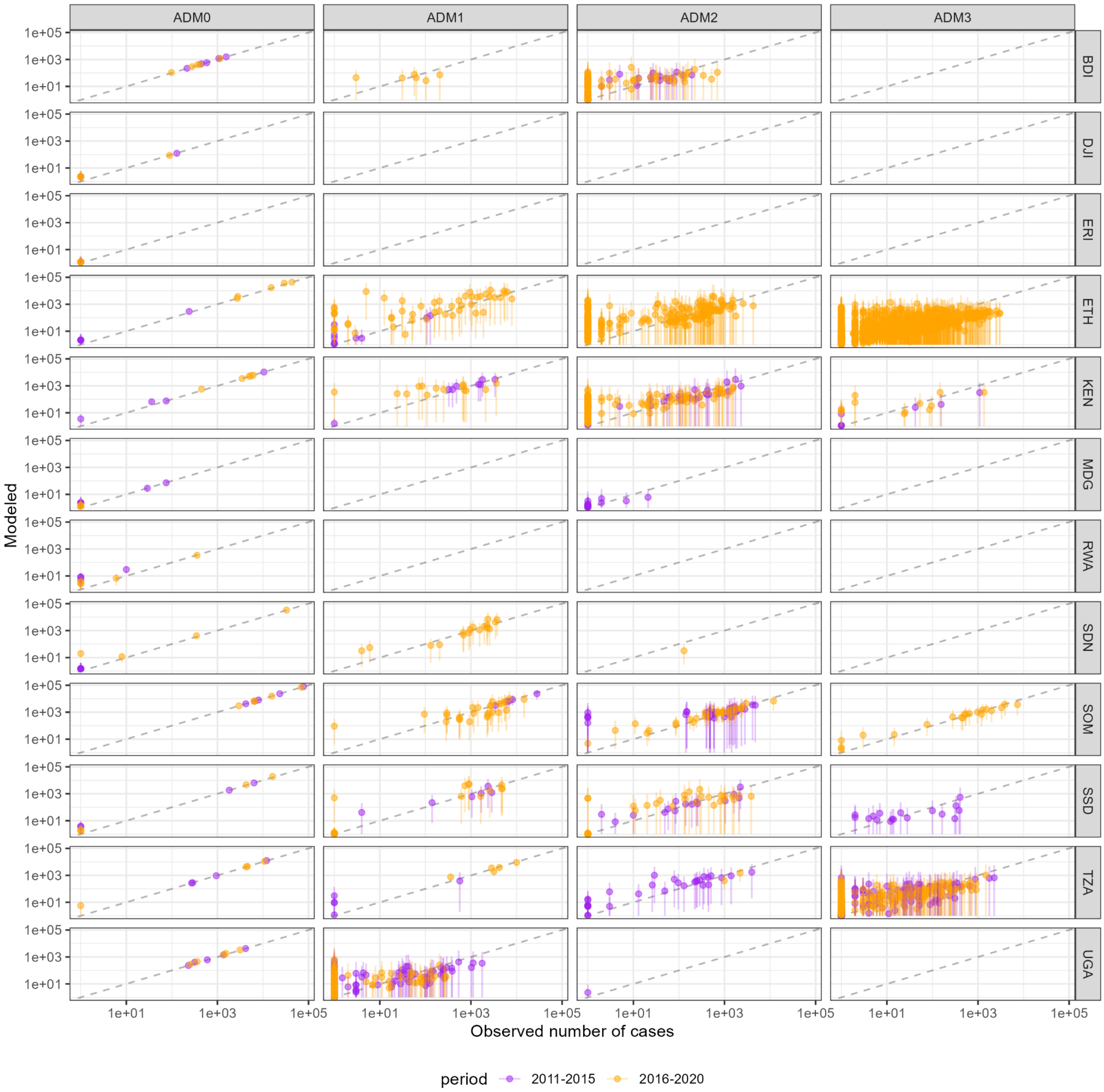

Figure S8. Scatterplot of full-year observations versus fitted values by administrative unit levels 0 to 3 for countries in Eastern Africa. Legend as in Figure S6.

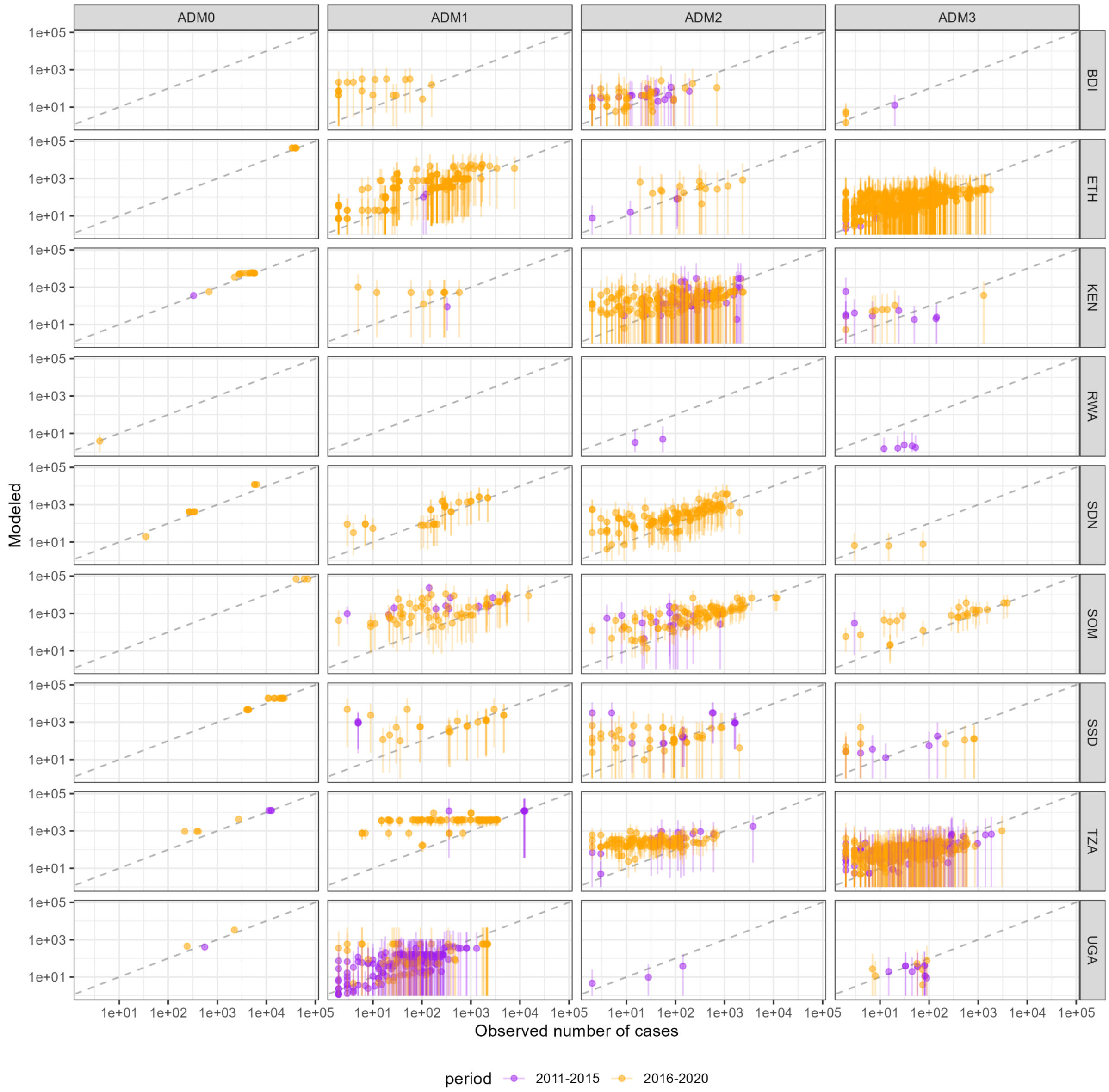

Figure S9. Scatterplot of partial-year observations versus fitted values by administrative unit levels 0 to 3 for countries in Eastern Africa. Legend as in Figure S7.

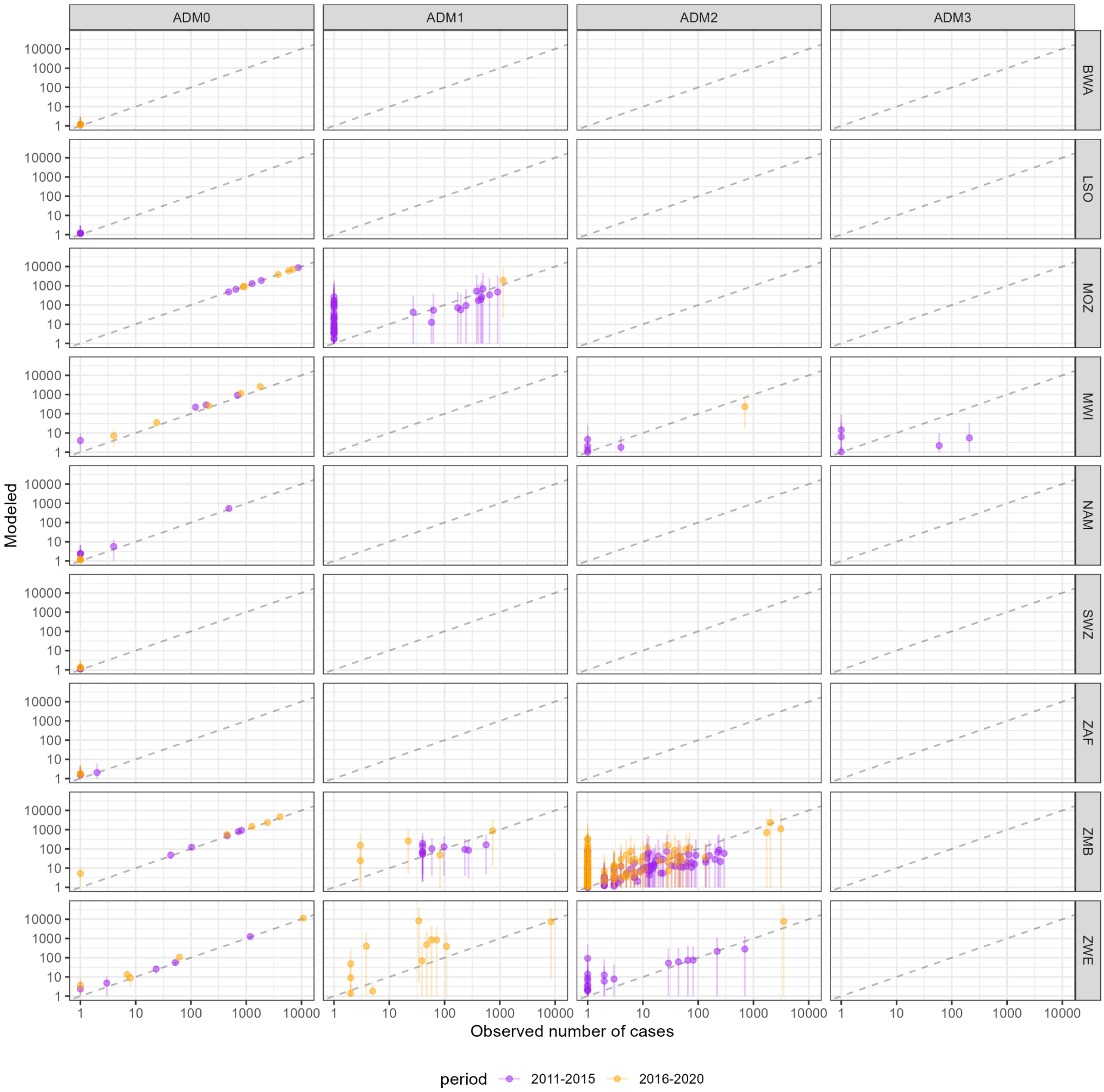

Figure S10. Scatterplot of full-year observations versus fitted values by administrative unit levels 0 to 3 for countries in Southern Africa. Legend as in Figure S6.

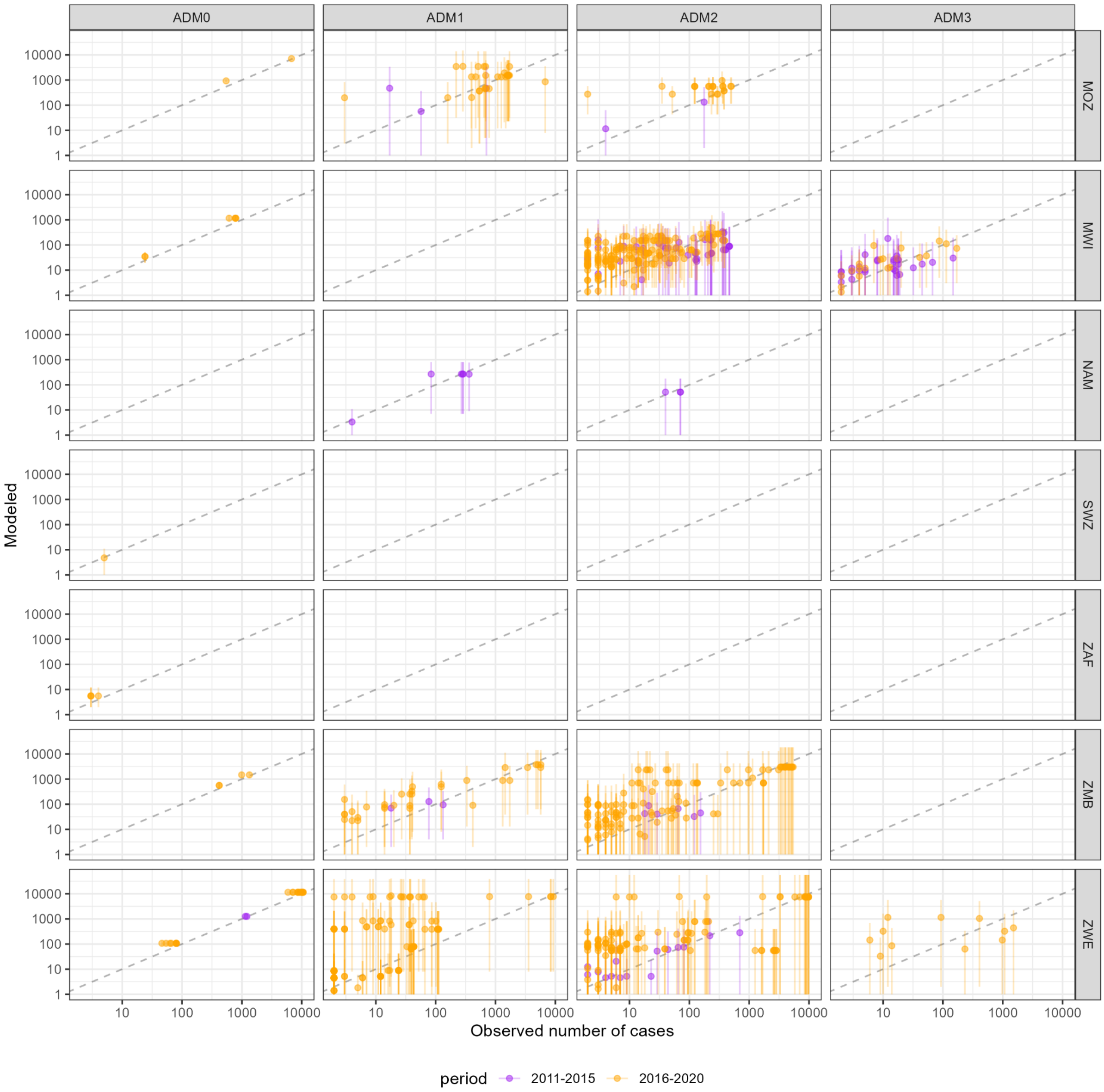

Figure S11. Scatterplot of partial-year observations versus fitted values by administrative unit levels 0 to 3 for countries in Southern Africa. Legend as in Figure S7.

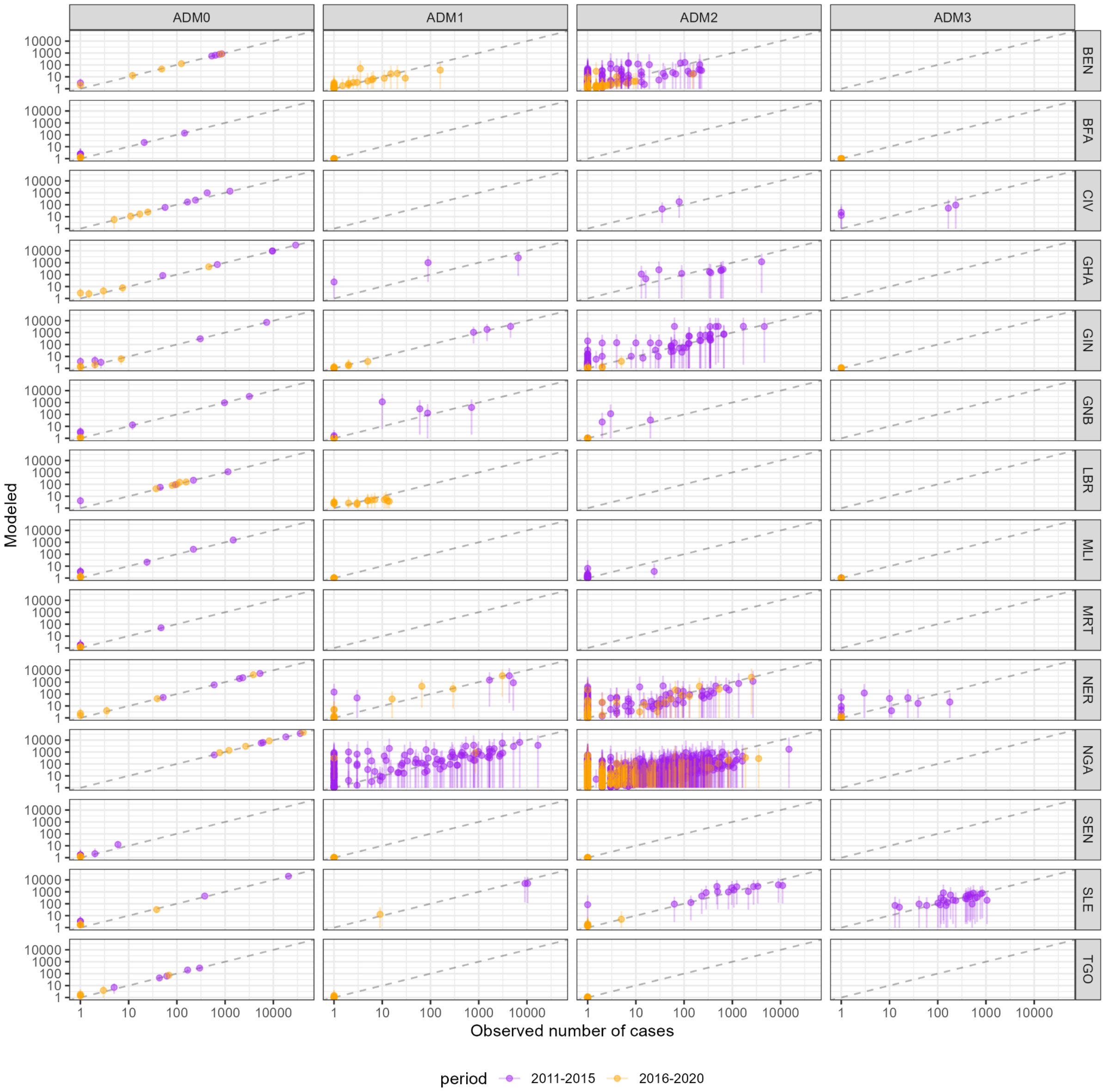

Figure S12. Scatterplot of full-year observations versus fitted values by administrative unit levels 0 to 3 for countries in Western Africa. Legend as in Figure S6.

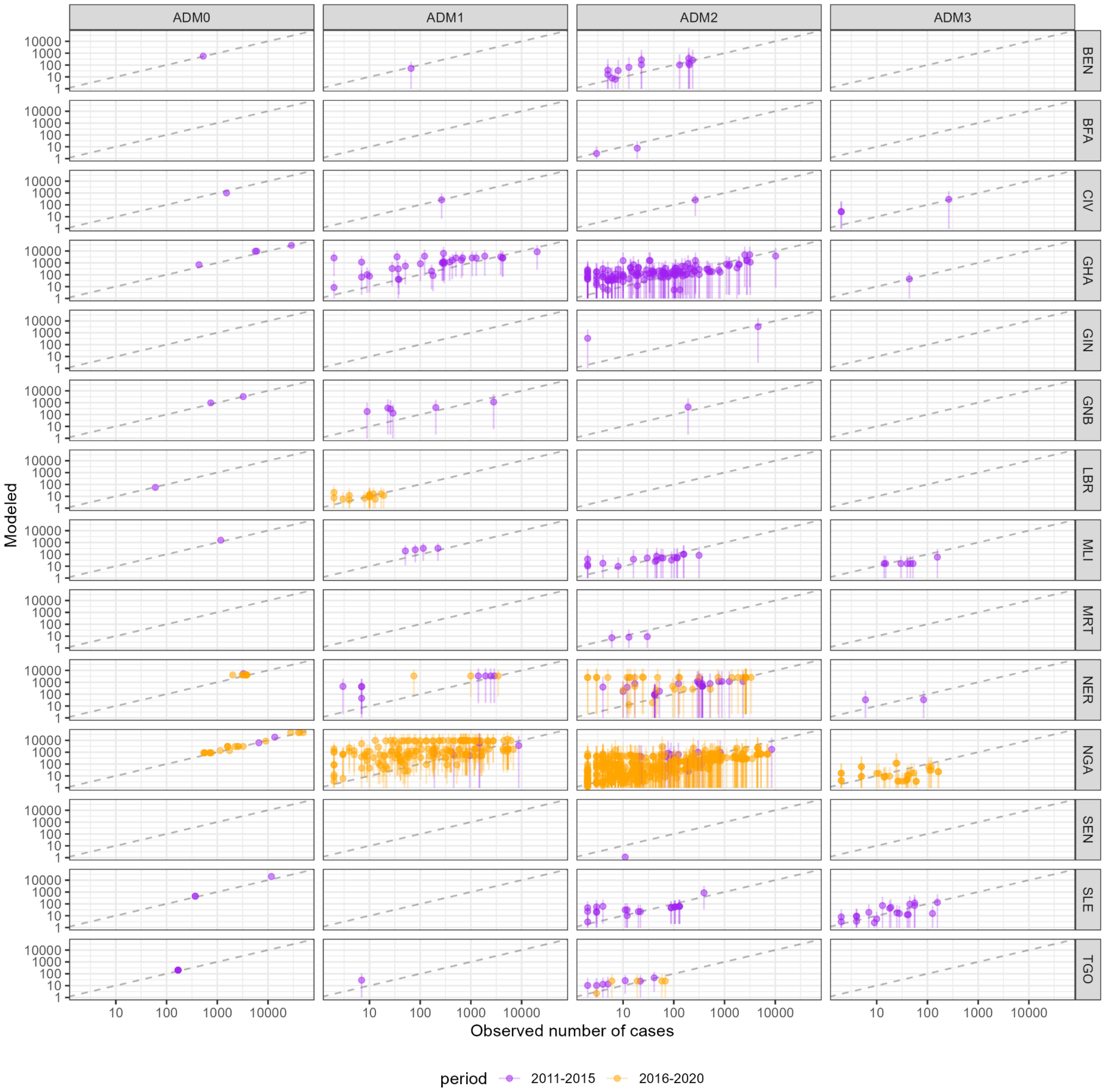

Figure S13. Scatterplot of partial-year observations versus fitted values by administrative unit levels 0 to 3 for countries in Western Africa. Legend as in Figure S7.

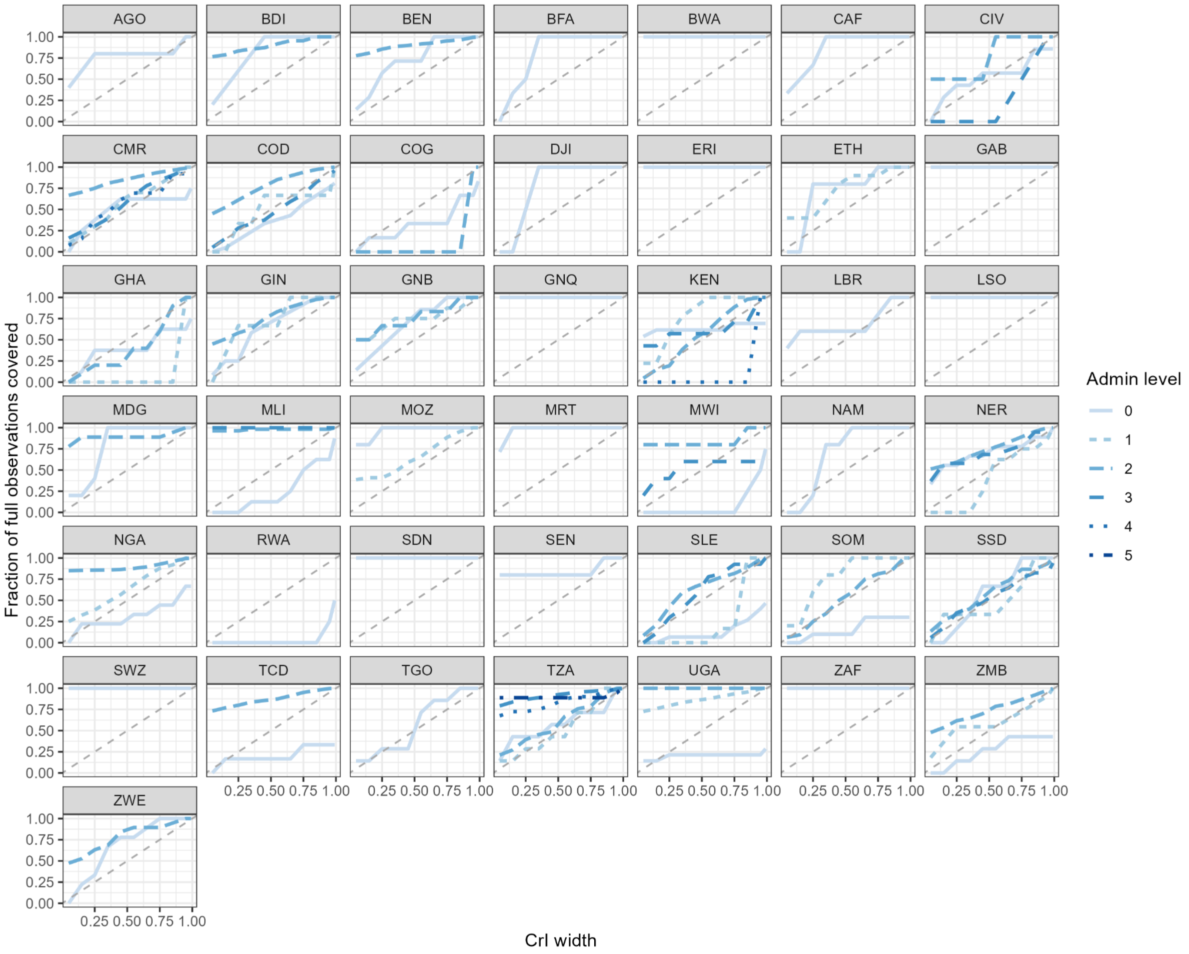

Figure S14. Coverage of full-year model observations by administrative unit level versus the credible interval width for 2011-2015 models. This figure indicates the appropriateness of the width of the fitted credible intervals based on the spread of the observation data. In a model that is neither overfit (with an interval that is too narrow) nor underfit (with an interval that is too wide), the credible interval width should roughly match the fraction of observations covered by the interval. For example, at the 50% CrI (x-axis), 50% of full observations (y-axis) should fall within the interval. Note that administrative unit level 0 (country-level) coverage is not comparable to that of other levels because the model fit is more restricted for these observations.

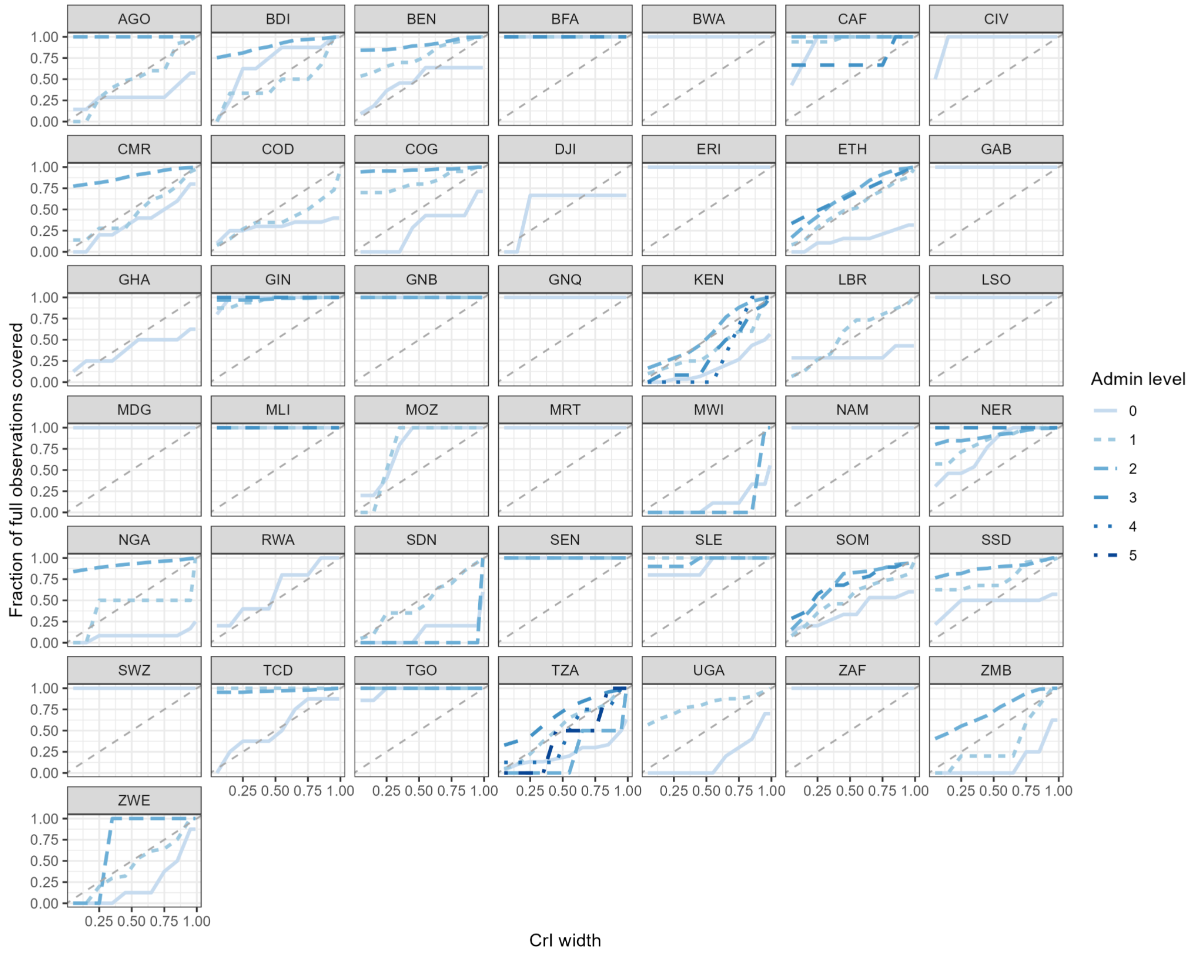

Figure S15. Coverage of full-year model observations by administrative unit level versus the credible interval width for 2016-2020 models. Legend as in Figure S14.

Supplementary Tables

Table S1. Summary of model observations by period. This table represents observation counts after data processing steps including temporal aggregation, but it excludes imputed national-level observations.

Period	Data sources	Countries	Locations	Administrative levels	Observations	National Observations	Subnational observations
2011-15	291	43	2,944	6	14,872	276	14,596
2016-20	622	43	3,473	7	15,230	587	14,643
2011-20	807	43	4,574	7	30,102	863	29,239

Table S2. Summary of full-year observations by period. This summary represents all model input observations, including imputed national-level observations. Observations with a time fraction greater than or equal to eight months (0.65 years) were considered full-year observations. Observations with shorter time fractions were considered as right-censored in the model likelihood.

Period	Full-year observations (%)	Total observations
2011-2015	13,324 (89)	14,940
2016-2020	8,756 (57)	15,271
2011-2020	22,080 (73)	30,211

Table S3. Number of sources and observations for all suspected case definitions by administrative unit level included in models.

No.	Case definition	Admin level	Number of sources	Number of observations
1	Any person presents with or dies from acute watery diarrhoea.	1	266	1521
2	Any person presents with or dies from acute watery diarrhoea.	2	228	6088
3	Any person presents with or dies from acute watery diarrhoea.	0	194	288
4	Unknown	0	133	498
5	Unknown	2	96	10022
6	Unknown	1	75	1016
7	Any person presents with or dies from acute watery diarrhoea.	3	66	1987
8	Unknown	3	24	2825
9	In a non-epidemic setting, a patient aged 5 years or more develops severe dehydration or dies from acute watery diarrhoea.	2	19	501
10	In an epidemic setting, a patient aged 2 years or more develops acute watery diarrhoea, with or without vomiting.	2	16	233
11	A patient aged 2 years or more develops acute watery diarrhoea, with or without vomiting.	1	14	860

12	A patient aged 2 years or more develops acute watery diarrhoea, with or without vomiting.	2	13	708
13	In a non-epidemic setting, a patient aged 5 years or more develops severe dehydration or dies from acute watery diarrhoea.	0	12	19
14	A patient aged 2 years or more develops acute watery diarrhoea, with or without vomiting.	3	11	294
15	In an epidemic setting, a patient aged 2 years or more develops acute watery diarrhoea, with or without vomiting.	1	11	186
16	A patient aged 2 years or more develops acute watery diarrhoea, with or without vomiting.	0	10	22
17	In a non-epidemic setting, a patient aged 5 years or more develops severe dehydration or dies from acute watery diarrhoea.	1	7	50
18	In a non-epidemic setting, a patient aged 5 years or more develops severe dehydration or dies from acute watery diarrhoea.	3	7	17
19	In an epidemic setting, a patient aged 2 years or more develops acute watery diarrhoea, with or without vomiting.	0	6	11
20	In an epidemic setting, a patient aged 2 years or more develops acute watery diarrhoea, with or without vomiting.	3	4	25
21	In a non-epidemic setting, a patient aged 5 years or more develops severe dehydration or dies from acute watery diarrhoea.	4	3	4
22	A patient aged 2 years or more develops acute watery diarrhoea, with or without vomiting.	4	2	25
23	Any person presents acute watery diarrhoea.	1	2	6
24	Any person presents acute watery diarrhoea.	2	2	34
25	A patient aged 5 years or more develops acute watery diarrhoea and severe dehydration, with or without vomiting. In an epidemic setting, a patient aged 2 years or more develops acute watery diarrhoea, with or without vomiting.	2	1	1790
26	A patient aged 5 years or more develops acute watery diarrhoea and severe dehydration, with or without vomiting. In an epidemic setting, a patient aged 2 years or more develops acute watery diarrhoea, with or without vomiting.	3	1	96

27	A patient aged 5 years or more develops acute watery diarrhoea, with or without vomiting and was hospitalized for at least 1 night and treated with intravenous fluids.	0	1	1
28	A patient aged 5 years or more develops acute watery diarrhoea, with or without vomiting.	0	1	1
29	A patient aged 5 years or more develops severe dehydration or dies from 3 or more acute watery stool per day, with or without vomiting. A patient aged 2-4 years develops severe dehydration or dies from acute watery diarrhoea, with or without vomiting.	1	1	8
30	A patient aged 5 years or more develops severe dehydration or dies from 3 or more acute watery stool per day, with or without vomiting. A patient aged 2-4 years develops severe dehydration or dies from acute watery diarrhoea, with or without vomiting.	2	1	12
31	A patient aged 5 years or more develops severe dehydration or dies from 3 or more acute watery stool per day, with or without vomiting. A patient aged 2-4 years develops severe dehydration or dies from acute watery diarrhoea, with or without vomiting.	3	1	1
32	Any patient develops acute watery nonbloody diarrhoea with more than 3 liquid stools in a day.	0	1	1
33	Any patient develops acute watery nonbloody diarrhoea with more than 3 liquid stools in a day.	1	1	3
34	Any patient develops acute watery nonbloody diarrhoea with more than 3 liquid stools in a day.	2	1	17
35	Any person aged 2 years or more presents with acute watery diarrhoea and severe dehydration or dies from acute watery diarrhoea.	1	1	7
36	Any person aged 2 years or more presents with acute watery diarrhoea and severe dehydration or dies from acute watery diarrhoea.	2	1	13
37	Any person aged 2 years or more presents with acute watery diarrhoea and severe dehydration or dies from acute watery diarrhoea.	3	1	33
38	Any person aged 2 years or more presents with acute watery diarrhoea and severe dehydration or dies from acute watery diarrhoea.	4	1	54
39	Any person aged 2 years or more presents with	5	1	22

	acute watery diarrhoea and severe dehydration or dies from acute watery diarrhoea.			
40	Any person presents with or dies from acute nonbloody watery diarrhoea with more than three liquid stools per day.	0	1	1
41	Any person presents with or dies from acute watery diarrhoea.	4	1	2
42	At the community level, any person presents with or dies from acute watery diarrhoea. At the health facility level and in a non-epidemic setting, a patient aged 5 years or more develops acute watery diarrhoea, with or without vomiting. At the health facility level and in an epidemic setting, a patient aged 2 years or more develops acute watery diarrhoea, with or without vomiting.	0	1	1
43	At the community level, any person presents with or dies from acute watery diarrhoea. At the health facility level and in a non-epidemic setting, a patient aged 5 years or more develops acute watery diarrhoea, with or without vomiting. At the health facility level and in an epidemic setting, a patient aged 2 years or more develops acute watery diarrhoea, with or without vomiting.	1	1	2
44	In a endemic setting, any person presents with acute watery diarrhoea.	0	1	1
45	In a endemic setting, any person presents with acute watery diarrhoea.	1	1	2
46	In a endemic setting, any person presents with acute watery diarrhoea.	2	1	12
47	In a non-epidemic setting, a patient aged 2 years or more develops acute watery diarrhoea and severe dehydration or death, with or without vomiting. In an epidemic setting, any person presents with or dies from acute watery diarrhoea.	0	1	16
48	In a non-epidemic setting, a patient aged 5 years or more develops severe dehydration or dies from acute watery diarrhoea. In an epidemic setting, a patient aged 5 years or more develops acute watery diarrhoea.	2	1	388
49	In a non-epidemic setting, any patient presents with servere dehydration or dies from acute watery diarrhoea.	1	1	1
50	In a non-epidemic setting, any patient presents with servere dehydration or dies from acute	2	1	2

	watery diarrhoea.			
51	In an non-endemic setting, a patient aged 5 years or more develops acute watery diarrhoea with severe dehydration or death, with or without vomiting. In an epidemic setting, any person presents 3 or more liquid stools with or without vomiting for the past 24 hours.	0	1	3
52	In an non-endemic setting, a patient aged 5 years or more develops acute watery diarrhoea with severe dehydration or death, with or without vomiting. In an epidemic setting, any person presents 3 or more liquid stools with or without vomiting for the past 24 hours.	2	1	59
53	In an non-epidemic setting, a patient aged 5 years or more develops acute watery diarrhoea and severe dehydration, with or without vomiting. In an epidemic setting, a patient aged 2 years or more develops acute watery diarrhoea, with or without vomiting.	2	1	301
54	Unknown	4	1	4
55	Unknown	6	1	8

Table S4. List of statistical model structures and data processing settings for each country and time period. Blank cells indicate that there was either no data processing deviation or no non-standard settings.

Country (ISO3 code)	Period	Model	Data processing deviation, if any	Reasons for non-standard settings
Angola (AGO	2011-2015	Non-mixture prior	-	Limited subnational data
	2016-2020	Non-mixture prior	-	Limited subnational data
Burundi (BDI	2011-2015	Standard	-	-
	2016-2020	Standard	-	-
Benin (BEN)	2011-2015	Standard	-	-
	2016-2020	Standard	-	-
Burkina Faso (BFA)	2011-2015	Non-mixture prior	-	Limited subnational data
	2016-2020	No spatial autoregressive term	-	Zero cases
Botswana (BWA)	2011-2015	No spatial autoregressive term	-	Zero cases
	2016-2020	No spatial	-	Zero cases

		autoregressive term		
Central African Republic (CAF)	2011-2015	Non-mixture prior	-	Limited subnational data
	2016-2020	Non-mixture prior	-	Limited subnational data
Côte d'Ivoire (CIV)	2011-2015	Non-mixture prior	-	Limited subnational data
	2016-2020	No spatial autoregressive term	-	No subnational data
Cameroon (CMR)	2011-2015	Standard	-	-
	2016-2020	Standard	-	-
Democratic Republic of the Congo (COD)	2011-2015	Standard	-	-
	2016-2020	Standard	Censoring threshold is 1	Substantial annual and incremental near-annual observations
Republic of the Congo (COG)	2011-2015	Standard	-	-
	2016-2020	Standard	-	-
Djibouti (DJI)	2011-2015	No spatial autoregressive term	-	No subnational data
	2016-2020	No spatial autoregressive term	-	No subnational data
Eritrea (ERI)	2011-2015	No spatial autoregressive term	-	Zero cases
	2016-2020	No spatial autoregressive term	-	Zero cases
Ethiopia (ETH)	2011-2015	Standard	-	-
	2016-2020	Standard		-
Gabon (GAB)	2011-2015	No spatial autoregressive term	-	Zero cases
	2016-2020	No spatial autoregressive term	-	Zero cases
Ghana (GHA)	2011-2015	Standard	-	-
	2016-2020	No spatial autoregressive term	-	No subnational data
Guinea (GIN)	2011-2015	Standard	-	-
	2016-2020	Standard	-	Limited subnational data

Guinea-Bissau (GNB	2011-2015	Non-mixture prior	-	Limited subnational data
	2016-2020	No spatial autoregressive term	-	Zero cases
Equatorial Guinea (GNQ)	2011-2015	No spatial autoregressive term	-	Zero cases
	2016-2020	No spatial autoregressive term	-	Zero cases
Kenya (KEN)	2011-2015	Standard	-	-
	2016-2020	Non-mixture prior	-	Improved convergence
Liberia (LBR)	2011-2015	No spatial autoregressive term	-	No subnational data
	2016-2020	Standard	-	-
Lesotho (LSO)	2011-2015	No spatial autoregressive term	-	Zero cases
	2016-2020	No spatial autoregressive term	-	Zero cases
Madagascar (MDG)	2011-2015	Non-mixture prior	-	Limited subnational data
	2016-2020	No spatial autoregressive term	-	Zero cases
Mali (MLI)	2011-2015	Non-mixture prior	-	Limited subnational data
	2016-2020	No spatial autoregressive term	-	Zero cases
Mozambique (MOZ)	2011-2015	Non-mixture prior	-	Limited subnational data
	2016-2020	Standard	-	-
Mauritania (MRT)	2011-2015	Non-mixture prior	-	Limited subnational data
	2016-2020	No spatial autoregressive term	-	Zero cases
Malawi (MWI	2011-2015	Standard	-	-
	2016-2020	Standard	-	-
Namibia (NAM)	2011-2015	Non-mixture prior	-	Limited subnational data
	2016-2020	No spatial autoregressive term	-	Zero cases
Niger (NER)	2011-2015	Standard	-	-

	2016-2020	Standard	-	-
Nigeria (NGA	2011-2015	Standard	-	-
	2016-2020	Standard	Censoring threshold is 1	Substantial annual and incremental near-annual observations
Rwanda (RWA)	2011-2015	Standard	-	-
	2016-2020	No spatial autoregressive term	-	Zero cases
Sudan (SDN)	2011-2015	No spatial autoregressive term	-	Zero cases
	2016-2020	Standard	-	-
Senegal (SEN)	2011-2015	No spatial autoregressive term	-	Limited subnational data
	2016-2020	No spatial autoregressive term	-	Zero cases
Sierra Leone (SLE)	2011-2015	Standard	-	-
	2016-2020	Non-mixture prior	-	Improved convergence
Somalia (SOM)	2011-2015	Non-mixture prior	-	Improved convergence
	2016-2020	Standard	-	-
South Sudan (SSD)	2011-2015	Standard	-	-
	2016-2020	Standard	-	-
Eswatini (SWZ)	2011-2015	No spatial autoregressive term	-	Zero cases
	2016-2020	No spatial autoregressive term	-	No subnational data
Chad (TCD)	2011-2015	Standard	-	-
	2016-2020	Standard	-	-
Togo (TGO)	2011-2015	Non-mixture prior	-	Limited subnational data
	2016-2020	Non-mixture prior	-	Limited subnational data
Tanzania (TZA)	2011-2015	Standard	-	-
	2016-2020	Standard	-	-
Uganda (UGA)	2011-2015	Standard	-	-
	2016-2020	Standard	-	-
South Africa (ZAF)	2011-2015	No spatial autoregressive term	-	Zero cases

	2016-2020	No spatial autoregressive term	-	No subnational data
Zambia (ZMB)	2011-2015	Standard	-	-
	2016-2020	Standard	-	-
Zimbabwe (ZWE)	2011-2015	Standard	-	-
	2016-2020	Standard	-	-

Table S5. Source of unified geographic shapefiles for modeled outputs at the country and second administrative level scales.

Source	Countries
GADM v4.1 (pulled from R package geodata v0.5.9)	Angola, Benin, Botswana, Burkina Faso, Cameroon, Central African Republic, Chad, Côte d'Ivoire, Djibouti, Equatorial Guinea, Eritrea, Eswatini, Gabon, Ghana, Guinea, Guinea-Bissau, Kenya, Lesotho, Liberia, Madagascar, Mali, Mauritania, Mozambique, Namibia, Niger, Nigeria, Republic of the Congo, Rwanda, Senegal, Sierra Leone, Somalia South Africa, South Sudan, Sudan, Tanzania, Togo, Zambia, Zimbabwe
geoboundaries v3.0 (pulled from R package rgeoboundaries v0.0.0.9)	Burundi, Democratic Republic of the Congo, Ethiopia, Malawi, Uganda

Table S6. Public documents from which cholera occurrence data were extracted in the post-2020 period by country, administrative unit level, and time range. Data from these documents were used for the analysis assessing associations between ten-year cholera incidence categories and post-2020 cholera occurrence. While the comprehensive time range represented in the data was from October 2021 to January 2024, the vast majority of data represents cholera occurrence from January 2022 to December 2023.

Document Name	Country	Admin-level and Time Range
WHO External Sitrep #1	Burundi	Admin 1: Jan 2023 - Mar 2023<br>Admin 2: Jan 2023 - Mar 2023
	Democratic Republic of the Congo	Admin 2: Jan 2023 - Mar 2023<br>Admin 3: Jan 2023 - Mar 2023
	Malawi	Admin 1: Mar 2023 - Mar 2023
	Mozambique	Admin 2: Sep 2022 - Mar 2023<br>Admin 3: Sep 2022 - Mar 2023
	South Africa	Admin 2: Jan 2023 - Mar 2023

	Tanzania	Admin 2: Jan 2023 - Mar 2023
	Zambia	Admin 2: Jan 2023 - Mar 2023<br>Admin 3: Jan 2023 - Mar 2023
	Zimbabwe	Admin 1: Jan 2023 - Mar 2023
WHO External Sitrep #2	Democratic Republic of the Congo	Admin 1: Jan 2023 - Apr 2023<br>Admin 2: Jan 2023 - Apr 2023
	Ethiopia	Admin 2: Jan 2023 - Jan 2023
	Kenya	Admin 1: Jan 2023 - Jan 2023
	Malawi	Admin 1: Apr 2023 - Apr 2023
	Somalia	Admin 2: Jan 2023 - Jan 2023
	Zimbabwe	Admin 1: Feb 2023 - Apr 2023
WHO External Sitrep #3	Cameroon	Admin 1: Mar 2023 - May 2023
	Democratic Republic of the Congo	Admin 2: Feb 2023 - Feb 2023
	Ethiopia	Admin 2: Feb 2023 - Feb 2023<br>Admin 3: Feb 2023 - Feb 2023
	Kenya	Admin 1: Feb 2023 - Feb 2023
	Malawi	Admin 1: Apr 2023 - May 2023
	Mozambique	Admin 2: Feb 2023 - May 2023
	Somalia	Admin 2: Feb 2023 - Feb 2023
	South Africa	Admin 2: Feb 2023 - Feb 2023
	Tanzania	Admin 2: Feb 2023 - Feb 2023
	Zambia	Admin 2: Feb 2023 - Feb 2023
	Zimbabwe	Admin 1: Feb 2023 - May 2023
WHO External Sitrep #4	Burundi	Admin 1: Mar 2023 - Mar 2023<br>Admin 2: Mar 2023 - Mar 2023
	Democratic Republic of the Congo	Admin 2: Mar 2023 - Mar 2023
	Eswatini	Admin 1: Mar 2023 - Mar 2023
	Ethiopia	Admin 2: Mar 2023 - Mar 2023<br>Admin 3: Mar 2023 - Mar 2023
	Kenya	Admin 1: Mar 2023 - Mar 2023
	Mozambique	Admin 2: Mar 2023 - Jun 2023<br>Admin 3: Mar 2023 - Mar 2023
	Malawi	Admin 1: May 2023 - Jun 2023
	Somalia	Admin 2: Mar 2023 - Mar 2023
	South Africa	Admin 2: Feb 2023 - Jun 2023
	Tanzania	Admin 1: Mar 2023 - Mar 2023<br>Admin 2: Mar 2023 - Mar 2023
	Zambia	Admin 2: Mar 2023 - Mar 2023

	Zimbabwe	Admin 1: Mar 2023 - Jun 2023
WHO External Sitrep #5	Burundi	Admin 2: Apr 2023 - Apr 2023
	Democratic Republic of the Congo	Admin 2: Apr 2023 - Apr 2023
	Eswatini	Admin 1: Apr 2023 - Apr 2023
	Ethiopia	Admin 2: Apr 2023 - Apr 2023<br>Admin 3: Apr 2023 - Apr 2023
	Kenya	Admin 1: Apr 2023 - Apr 2023
	Mozambique	Admin 2: Apr 2023 - Apr 2023<br>Admin 3: Apr 2023 - Apr 2023
	Malawi	Admin 1: Apr 2023 - Jul 2023
	Somalia	Admin 2: Apr 2023 - Apr 2023
	South Africa	Admin 2: Feb 2023 - Jul 2023
	Zambia	Admin 2: Apr 2023 - Apr 2023
	Zimbabwe	Admin 1: Apr 2023 - Apr 2023
WHO External Sitrep #6	Burundi	Admin 2: May 2023 - May 2023
	Democratic Republic of the Congo	Admin 2: May 2023 - May 2023
	Ethiopia	Admin 2: May 2023 - May 2023<br>Admin 3: May 2023 - May 2023
	Kenya	Admin 1: May 2023 - May 2023
	Mozambique	Admin 1: May 2023 - May 2023<br>Admin 2: May 2023 - Aug 2023
	Malawi	Admin 1: May 2023 - Aug 2023
	Somalia	Admin 2: May 2023 - May 2023
	South Africa	Admin 2: May 2023 - May 2023
	Tanzania	Admin 1: May 2023 - May 2023<br>Admin 2: May 2023 - May 2023
	Zambia	Admin 2: May 2023 - May 2023
	Zimbabwe	Admin 1: May 2023 - May 2023<br>Admin 2: May 2023 - May 2023
WHO External Sitrep #7	Burundi	Admin 2: Dec 2022 - Sep 2023
	Democratic Republic of the Congo	Admin 2: Jun 2023 - Jun 2023
	Ethiopia	Admin 2: Jun 2023 - Jun 2023<br>Admin 3: Jun 2023 - Jun 2023
	Kenya	Admin 1: Jun 2023 - Jun 2023
	Mozambique	Admin 2: May 2023 - Sep 2023<br>Admin 3: Jun 2023 - Jun 2023
	Malawi	Admin 1: Jun 2023 - Jun 2023
	Somalia	Admin 2: Jun 2023 - Jun 2023

		Admin 3: Jun 2023 - Jun 2023
	South Africa	Admin 1: Jun 2023 - Jun 2023
	Zambia	Admin 2: Jun 2023 - Jun 2023
	Zimbabwe	Admin 1: Jun 2023 - Jun 2023
WHO External Sitrep #8	Burundi	Admin 2: Jul 2023 - Jul 2023
	Democratic Republic of the Congo	Admin 2: Jul 2023 - Jul 2023
	Ethiopia	Admin 2: Jul 2023 - Jul 2023<br>Admin 3: Jul 2023 - Jul 2023
	Kenya	Admin 1: Jul 2023 - Jul 2023<br>Admin 3: Jul 2023 - Jul 2023
	Mozambique	Admin 2: Jul 2023 - Oct 2023<br>Admin 3: Jul 2023 - Oct 2023
	Malawi	Admin 1: Jul 2023 - Jul 2023
	Somalia	Admin 2: Jul 2023 - Jul 2023<br>Admin 3: Jul 2023 - Jul 2023
	South Africa	Admin 2: Jul 2023 - Jul 2023<br>Admin 3: Jul 2023 - Jul 2023
	Tanzania	Admin 1: Jul 2023 - Jul 2023<br>Admin 2: Jul 2023 - Jul 2023
	Uganda	Admin 1: Jul 2023 - Jul 2023
	Zimbabwe	Admin 1: Jul 2023 - Oct 2023
WHO External Sitrep #9	Burundi	Admin 2: Aug 2023 - Aug 2023
	Democratic Republic of the Congo	Admin 2: Aug 2023 - Aug 2023
	Ethiopia	Admin 1: Aug 2023 - Aug 2023
	Kenya	Admin 1: Aug 2023 - Aug 2023
	Mozambique	Admin 2: Aug 2023 - Nov 2023<br>Admin 3: Aug 2023 - Nov 2023
	Malawi	Admin 1: Aug 2023 - Aug 2023
	Somalia	Admin 2: Aug 2023 - Aug 2023<br>Admin 3: Aug 2023 - Aug 2023
	Sudan	Admin 1: Aug 2023 - Aug 2023
	Uganda	Admin 1: Aug 2023 - Aug 2023<br>Admin 2: Aug 2023 - Aug 2023
	Zambia	Admin 2: Aug 2023 - Aug 2023
	Zimbabwe	Admin 1: Aug 2023 - Nov 2023
WHO External Sitrep #10	Burundi	Admin 1: Sep 2023 - Sep 2023<br>Admin 2: Sep 2023 - Dec 2023
	Democratic Republic of the Congo	Admin 2: Sep 2023 - Jan 2024<br>Admin 3: Oct 2023 - Dec 2023
	Ethiopia	Admin 1: Sep 2023 - Dec 2023

	Kenya	Admin 1: Sep 2023 - Nov 2023
	Mozambique	Admin 2: Sep 2023 - Dec 2023<br>Admin 3: Sep 2023 - Dec 2023
	Malawi	Admin 1: Sep 2023 - Dec 2023
	Somalia	Admin 2: Sep 2023 - Dec 2023
	Sudan	Admin 1: Sep 2023 - Dec 2023
	Tanzania	Admin 1: Oct 2023 - Dec 2023
	Zambia	Admin 2: Sep 2023 - Dec 2023
	Zimbabwe	Admin 1: Sep 2023 - Dec 2023
AFRO Cholera Bulletin.49	Cameroon	Admin 1: Oct 2021 - Jan 2024
	Togo	Admin 1: Dec 2023 - Dec 2023
	Zambia	Admin 1: Jan 2024 - Jan 2024
An update of Cholera outbreak in Nigeria_221222_52	Nigeria	Admin 1: Jan 2022 - Dec 2022
An update of Cholera outbreak in Nigeria_221223_52	Nigeria	Admin 1: Jan 2023 - Dec 2023
Weekly Bulletin on Outbreaks and Other Emergencies - WHO African Region	Tanzania	Admin 2: Sep 2023 - Oct 2023
South Sudan Cholera Situation Report_Issue #39	South Sudan	Admin 2: Feb 2023 - Apr 2023
WHO Sudan Outbreaks dashboard	Sudan	Admin 3: Jun 2023 - Jan 2024
