# Supplementary material for: Geographical shifting of cholera burden in Africa and its implications for disease control
Source: Nat Med. 2025 Aug 7;31(10):3380–7. doi: 10.1038/s41591-025-03847-9 (PMC12532610; doi:10.1038/s41591-025-03847-9)
Supplement: Supplementary file 2 — Reporting Summary [file 41591_2025_3847_MOESM2_ESM.pdf]

nature portfolio

Corresponding author(s): Elizabeth Lee

Last updated by author(s): Jun 7, 2025

Reporting Summary

Nature Portfolio wishes to improve the reproducibility of the work that we publish. This form provides structure for consistency and transparency in reporting. For further information on Nature Portfolio policies, see our Editorial Policies and the Editorial Policy Checklist.

Statistics

For all statistical analyses, confirm that the following items are present in the figure legend, table legend, main text, or Methods section.

n/a	Confirmed	
☐	☒	The exact sample size (n) for each experimental group/condition, given as a discrete number and unit of measurement
☒	☐	A statement on whether measurements were taken from distinct samples or whether the same sample was measured repeatedly
☐	☒	The statistical test(s) used AND whether they are one- or two-sided<br>*Only common tests should be described solely by name; describe more complex techniques in the Methods section.*
☐	☒	A description of all covariates tested
☐	☒	A description of any assumptions or corrections, such as tests of normality and adjustment for multiple comparisons
☐	☒	A full description of the statistical parameters including central tendency (e.g. means) or other basic estimates (e.g. regression coefficient) AND variation (e.g. standard deviation) or associated estimates of uncertainty (e.g. confidence intervals)
☒	☐	For null hypothesis testing, the test statistic (e.g. F, t, r) with confidence intervals, effect sizes, degrees of freedom and P value noted<br>*Give P values as exact values whenever suitable.*
☐	☒	For Bayesian analysis, information on the choice of priors and Markov chain Monte Carlo settings
☐	☒	For hierarchical and complex designs, identification of the appropriate level for tests and full reporting of outcomes
☒	☐	Estimates of effect sizes (e.g. Cohen's d, Pearson's r), indicating how they were calculated

Our web collection on statistics for biologists contains articles on many of the points above.

Software and code

Policy information about availability of computer code

Data collection	A custom database was used to store and make public cholera incidence data, and the front-end may be viewed at https://cholera-taxonomy.middle-distance.com/
Data analysis	Data processing and modeling code is publicly available with a GPLv3 license on Github at https://github.com/HopkinsIDD/cholera-mapping-pipeline, release v1.1. Statistical modeling and figure generation were performed on R version 4.0.3.

For manuscripts utilizing custom algorithms or software that are central to the research but not yet described in published literature, software must be made available to editors and reviewers. We strongly encourage code deposition in a community repository (e.g. GitHub). See the Nature Portfolio guidelines for submitting code & software for further information.

Data

Policy information about availability of data

All manuscripts must include a data availability statement. This statement should provide the following information, where applicable:

- Accession codes, unique identifiers, or web links for publicly available datasets
- A description of any restrictions on data availability
- For clinical datasets or third party data, please ensure that the statement adheres to our policy

Cholera incidence datasets derived from public sources may be viewed and accessed from https://cholera-taxonomy.middle-distance.com with no restrictions while the database is maintained, at minimum for three years post-publication. Metadata for non-public cholera incidence datasets may be requested from the

corresponding author with a projected two-week turnaround while the database is maintained and a four-week turnaround after the database has been archived. Non-public incidence datasets will not be shared, in concordance with data sharing agreements. Spatial population distributions were obtained from the WorldPop global unconstrained mosaic population counts product (https://www.worldpop.org) and country-level population estimates were obtained from the United Nations Population Division World Population Prospects 2022. Underlying maps for the Democratic Republic of the Congo, Burundi, Ethiopia, Malawi, and Uganda were obtained from geoBoundaries, which has a CC BY 4.0 license. All other underlying country maps were obtained from GADM, which has a license allows for open access academic publishing. Gridded, ADM2, country, and region-level modeled outputs are available on Open Science Framework at https://osf.io/jzquw/ with no restrictions.

Research involving human participants, their data, or biological material

Policy information about studies with human participants or human data. See also policy information about sex, gender (identity/presentation), and sexual orientation and race, ethnicity and racism.

Reporting on sex and gender	No sex- and gender-based analyses have been performed because these groupings were not generally available in the publichealth surveillance data used for estimating disease burden.
Reporting on race, ethnicity, or other socially relevant groupings	N/A
Population characteristics	N/A
Recruitment	N/A
Ethics oversight	The Institutional Review Board (IRB) at Johns Hopkins Bloomberg School of Public Health (BSPH) determined that secondary analysis of data from the global cholera incidence database was exempt (BSPH IRB No. 27682) and no other institutional approvals were sought.

Note that full information on the approval of the study protocol must also be provided in the manuscript.

Field-specific reporting

Please select the one below that is the best fit for your research. If you are not sure, read the appropriate sections before making your selection.

☒ Life sciences ☐ Behavioural & social sciences ☐ Ecological, evolutionary & environmental sciences

For a reference copy of the document with all sections, see nature.com/documents/nr-reporting-summary-flat.pdf

Life sciences study design

All studies must disclose on these points even when the disclosure is negative.

Sample size	No sample size calculations were performed as there was no primary data collection or sampling design in our study.
Data exclusions	Data that could not be linked to recognizable geographic locations or valid geographic shapefiles (vector files) were excluded from the analysis.
Replication	Model results were determined to be reproducible given a fixed random seed during model testing. 4000 posterior estimates were generated for each model to develop credible interval estimates displayed in the paper.
Randomization	Randomization is not relevant to our study as there was no experimental intervention design and no primary data collection. We performed a secondary data analysis to estimate disease incidence with previously collected public health surveillance data. Population was used as a covariate in our analyses to estimate mean annual incidence rate.
Blinding	Blinding is not relevant to our study as there was no experimental intervention design and no primary data collection. We performed a secondary data analysis with previously collected public health surveillance data.

Reporting for specific materials, systems and methods

We require information from authors about some types of materials, experimental systems and methods used in many studies. Here, indicate whether each material, system or method listed is relevant to your study. If you are not sure if a list item applies to your research, read the appropriate section before selecting a response.

Materials & experimental systems

n/a	Involved in the study
☒	☐ Antibodies
☒	☐ Eukaryotic cell lines
☒	☐ Palaeontology and archaeology
☒	☐ Animals and other organisms
☒	☐ Clinical data
☒	☐ Dual use research of concern
☒	☐ Plants

Methods

n/a	Involved in the study
☒	☐ ChIP-seq
☒	☐ Flow cytometry
☒	☐ MRI-based neuroimaging

Plants

Seed stocks	*Report on the source of all seed stocks or other plant material used. If applicable, state the seed stock centre and catalogue number. If plant specimens were collected from the field, describe the collection location, date and sampling procedures.*
Novel plant genotypes	*Describe the methods by which all novel plant genotypes were produced. This includes those generated by transgenic approaches, gene editing, chemical/radiation-based mutagenesis and hybridization. For transgenic lines, describe the transformation method, the number of independent lines analyzed and the generation upon which experiments were performed. For gene-edited lines, describe the editor used, the endogenous sequence targeted for editing, the targeting guide RNA sequence (if applicable) and how the editor was applied.*
Authentication	*Describe any authentication procedures for each seed stock used or novel genotype generated. Describe any experiments used to assess the effect of a mutation and, where applicable, how potential secondary effects (e.g. second site T-DNA insertions, mosiacism, off-target gene editing) were examined.*
